# Identification of residues critical for the extension of Munc18-1 domain 3a

**DOI:** 10.1186/s12915-023-01655-6

**Published:** 2023-07-13

**Authors:** Xianping Wang, Jihong Gong, Le Zhu, Huidan Chen, Ziqi Jin, Xiaoqiang Mo, Shen Wang, Xiaofei Yang, Cong Ma

**Affiliations:** 1 Hubei Key Laboratory of Edible Wild Plants Conservation and Utilization, College of Life Sciences, Hubei Normal University, Huangshi, China; 2 Key Laboratory of Cognitive Science, Hubei Key Laboratory of Medical Information Analysis and Tumor Diagnosis & Treatment, Laboratory of Membrane Ion Channels and Medicine, College of Biomedical Engineering, South-Central Minzu University, Wuhan, China; 3 Key Laboratory of Molecular Biophysics of the Ministry of Education, College of Life Science and Technology, Huazhong University of Science and Technology, Wuhan, China; 4 Youjiang Medical University for Nationalities, Baise, China

**Keywords:** Munc18-1, Syntaxin-1, Munc13-1, SNARE complex, Conformational change, Synaptic exocytosis

## Abstract

**Background:**

Neurotransmitter release depends on the fusion of synaptic vesicles with the presynaptic membrane and is mainly mediated by SNARE complex assembly. During the transition of Munc18-1/Syntaxin-1 to the SNARE complex, the opening of the Syntaxin-1 linker region catalyzed by Munc13-1 leads to the extension of the domain 3a hinge loop, which enables domain 3a to bind SNARE motifs in Synaptobrevin-2 and Syntaxin-1 and template the SNARE complex assembly. However, the exact mechanism of domain 3a extension remains elusive.

**Results:**

Here, we characterized residues on the domain 3a hinge loop that are crucial for the extension of domain 3a by using biophysical and biochemical approaches and electrophysiological recordings. We showed that the mutation of residues T323/M324/R325 disrupted Munc13-1-mediated SNARE complex assembly and membrane fusion starting from Munc18-1/Syntaxin-1 in vitro and caused severe defects in the synaptic exocytosis of mouse cortex neurons in vivo. Moreover, the mutation had no effect on the binding of Synaptobrevin-2 to isolated Munc18-1 or the conformational change of the Syntaxin-1 linker region catalyzed by the Munc13-1 MUN domain. However, the extension of the domain 3a hinge loop in Munc18-1/Syntaxin-1 was completely disrupted by the mutation, leading to the failure of Synaptobrevin-2 binding to Munc18-1/Syntaxin-1.

**Conclusions:**

Together with previous results, our data further support the model that the template function of Munc18-1 in SNARE complex assembly requires the extension of domain 3a, and particular residues in the domain 3a hinge loop are crucial for the autoinhibitory release of domain 3a after the MUN domain opens the Syntaxin-1 linker region.

**Supplementary Information:**

The online version contains supplementary material available at 10.1186/s12915-023-01655-6.

## Background

Neurotransmitter release triggered by Ca^2+^ influx [1] is exquisitely regulated by multiple processes involving vesicles (1) docked to the active zone, (2) primed to a ready-for-release state, and (3) fused with the presynaptic membrane [2–4]. The core machinery is SNARE proteins, including Syntaxin-1 (Syx1) and SNAP-25 (SN25) on the presynaptic membranes (also known as t-SNAREs) and Synaptobrevin-2 (Syb2) on the synaptic vesicles (also called v-SNARE) [5–7]. The three SNAREs form the SNARE complex via the assembly of ~ 65-residue SNARE motifs into a four-helix bundle, which releases energy to bring the two membranes into close proximity and ultimately leads to fusion that occurs on the millisecond timescale [8–10]. The priming process is crucial for fusion and is characterized as the assembly of SNAREs into the SNARE complex, which requires numerous other components to achieve strict temporal and spatial regulation, such as the Sec1/Munc18-like (SM) protein Munc18-1 (M18-1), CATCHR (*c*omplexes *a*ssociated with *t*ethering *c*ontaining *h*elical *r*ods) family protein Munc13, synaptotagmins, and complexin [11–14].

Munc18-1, mainly expressed in mammalian neurons and neuroendocrine cells, plays a crucial role in vesicle docking, priming, and fusion [15–17]. Deficiency of Munc18-1 results in complete abrogation of neurotransmitter release in neurons and severely impairs docking of DCVs (dense core vesicles) in adrenal chromaffin cells [18, 19]. Several studies have revealed that Munc18-1 strictly controls SNARE complex assembly through interaction with different SNAREs with diverse conformations [17, 20]. Munc18-1 binds to Syx1 via its domains 1 and 3a, forming an arch-shaped architecture, locking Syx1 in a “closed conformation” with the Syx1 Habc domain folding back to form an intramolecular interaction with H3 [21, 22], which precludes the trapped Syx1 from assembling the SNARE complex. Notably, the inhibitory binding mode allows Munc18-1 to chaperone Syx1 to the presynaptic membrane accurately and vesicles to accumulate around the plasma membrane [23, 24]. When activated by Munc13-1, Syx1 loosens its closed conformation [25]. Subsequently, Munc18-1 is suggested as a platform to bind Syx1 and Syb2 simultaneously, orchestrating the SNARE complex assembly and promoting vesicle priming [26–29]. This binding mode allows the correct register and orientation of SNARE motifs on Munc18-1 domain 3a for efficient nucleation of SNARE assembly, which has been confirmed by the cryo-EM structure of Syb2/Munc18-1/Syx1 [28] and crystal structure of Vps33 (homologous with Munc18-1) bound to Nyv1 (homologous with Syb2) or Vam3 (homologous with Syx1) from the thermophilic fungus [30].

Increasing evidence has reinforced the validity of the model that Munc18-1 and Munc13-1 collaboratively organize SNARE complex assembly [31–34]. Neurotransmitter release is completely eliminated in *Munc18-1-* or *Munc13*-null mammalian neurons and *unc13*-null *Caenorhabditis elegans* (*C. elegans*), indicating the fundamental role of both proteins in regulating synaptic exocytosis [18, 35–38]. The transition from the Munc18-1/Syx1 complex rather than from the Syx1/SNAP-25 (t-SNARE complex) to the SNARE complex, activated by Munc13-1, indicates the central functions of Munc18-1 and Munc13-1 in promoting SNARE complex assembly and preventing the half-assembled *trans*-SNARE complex from being disassembled by NSF and α-SNAP [31, 33]. The transition involves at least two conformational changes: a “closed-to-open” change in the Syx1 linker region and a “bent-to-extended” change in Munc18-1 domain 3a [25, 39–41]. The “closed” and “bent” configurations in closed Munc18-1/Syx1 establish a series of energy barriers to hinder SNARE assembly and act as pivotal points for regulation by Munc13-1 and other components.

Our previous studies have revealed the relationship between the two conformational changes. The opening of the linker region leads to the extension of domain 3a but not vice versa, and both conformational changes depend on the interaction between Munc18-1/Syx1 and the Munc13-1 MUN domain [25, 29, 42]. Two gain-of-function mutations of L165A/E166A (LEAA) on Syx1, which helps to open the linker region, and the P335A mutation on Munc18-1, which facilitates domain 3a extension, partially rescue secretion in *unc13*-null *C. elegans* and support SNARE complex assembly with no requirement for Munc13-1 in vitro [35, 43]. In addition, another gain-of-function mutation, D326K, unfurling the domain 3a hinge loop and increasing the binding affinity between Munc18-1 and Syb2, showed an increased effect in rescuing synaptic transmission [27]. These findings raise the question of whether the conformation change is controlled by other residues in the hinge loop.

We and other groups have identified several residues in Munc18-1 domain 3a that are responsible for interactions with Syb2 (L348) [41], Syx1 H3 (K332/K333), MUN domain (Q301/K308), and SNARE complex (Y337) [29, 44], or the mutations that favor the extension of domain 3a (D326K and P335A) [27, 41]. Considering the importance of domain 3a, whether other residues are involved in regulating SNARE complex assembly and synaptic exocytosis remains elusive. To further shed light on the regulatory mechanism of the conformational change in Munc18-1 domain 3a, we generated several mutants to search for pivotal residues. In the present study, we identified three residues (T323/M324/R325) that are crucial for Munc18-1 to regulate SNARE complex assembly and neurotransmitter release. The ternary mutation (T323A/M324A/R325A) completely abolished the MUN-catalyzed transition from Munc18-1/Syx1 to the SNARE complex in vitro and severely impaired synaptic vesicle priming and fusion in vivo, but exerted no effects on the chaperoning of Syx1 to the plasma membrane in HEK293T cells, the interaction between Syb2 and isolated Munc18-1. Further single-molecule FRET (smFRET) experiments demonstrated that the mutant enabled the Syx1 linker region to be opened by the MUN domain. Interestingly, the mutation selectively hindered the formation of the quaternary complex Munc18-1/Syx1/Syb2/MUN. Taken together, these results reinforced the importance of residues T323/M324/R325 in regulating the conformational extension of domain 3a.

## Results

### Mutation T323A/M324A/R325A in the domain 3a hinge loop impaired the transition from Munc18-1/Syx1 to the SNARE complex

**Fig. 1:**
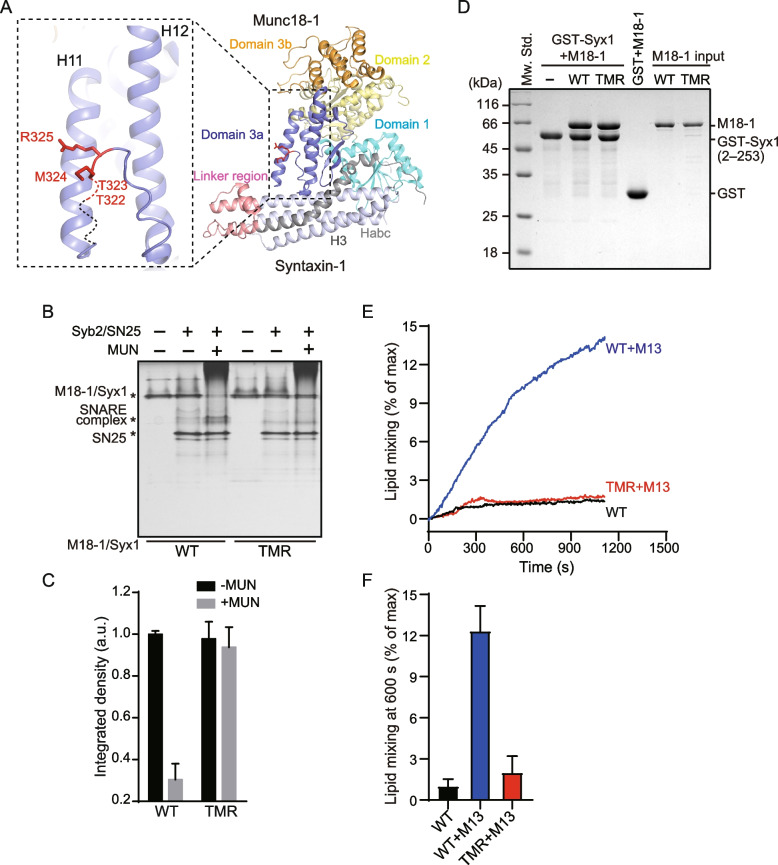
Residues T323/M324/R325 are essential for SNARE complex assembly from Munc18-1/Syx1 catalyzed by the MUN domain.** A** Crystal structure of Munc18-1/Syx1 (PDB ID: 3C98) and positions indicating mutation sites (T322, T323, M324 and R325). Mutation sites are highlighted in red, and the residue sequence (317–323) without electron density is shown as dashed lines. **B** The triple mutation T323A/M324A/R325A (TMR) completely abolished the transition from Munc18-1/Syx1 to the SNARE complex catalyzed by the MUN domain, as shown by native PAGE. **C** Quantification of the results shown in **B**. Data are presented as mean values ± SD; *n* = 3. **D** The TMR mutation had no effect on the interaction between Munc18-1 and Syx1, as detected by GST pull-down assay. **E** Effect of TMR mutation on fusion between liposomes containing Munc18-1/Syx1 and liposomes reconstituted with Syb2 in the presence of SNAP-25, the Syt1 C2AB fragment, and 1 mM CaCl_2_, with or without the Munc13-1 C1C2BMUN fragment. **F** The quantification results of data shown in **E**. Data are presented as mean values ± SD; *n* = 3. Representative gel displayed is from one of three independent replicates. M18-1, Munc18-1; Syx1, Syntaxin-1; M13, Munc13-1 C1C2BMUN domain; SN25, SNAP-25; Syb2, Synaptobrevin-2

Previous studies have demonstrated the functional importance of Munc18-1 domain 3a in regulating SNARE complex assembly, in which the template function depends on the conformational change of domain 3a and has attracted much attention in recent years [30, 45–47]. We have revealed that the extension of domain 3a occurs following the opening of the Syx1 linker region. However, the particular regulatory mechanism of the conformational change in domain 3a remains unclear. To further explore the specific residues that are crucial for the conformational change in domain 3a, we rationally designed mutations in the domain 3a hinge loop based on the crystal structure of Munc18-1/Syx1 (Fig. 1A). By using a previously described native PAGE assay [25, 48], the T323A, M324A, and R325A mutations were found to impair the transition from Munc18-1/Syx1 to the SNARE complex to various degrees in the presence of the MUN domain, Syb2, and SNAP-25. In contrast, the mutation T322A showed similar activity to wild-type (WT) Munc18-1 in the transition (Additional file 1: Fig. S1). Next, a ternary mutation T323A/M324A/R325A (TMR) was generated and completely hindered the transition, as shown by native gel electrophoresis (Fig. 1B, C).

To exclude the confounding effect caused by protein misfolding, size-exclusion chromatography was used to characterize all Munc18-1 proteins. All Munc18-1 mutants showed similar elution volumes to Munc18-1 WT, regardless of whether they were isolated or in complex with Syx1 (Additional file 1: Fig. S2). We suspected that the mutations interfere with SNARE complex assembly by affecting the binding affinity between Munc18-1 and Syx1. Thus, we performed a GST pull-down assay to test the interaction between Munc18-1 and Syx1 directly. The TMR mutation showed similar binding affinity to WT Munc18-1 (Fig. 1D). These results suggest that these mutations have no significant influence on the global conformation of Munc18-1 and that the inability is unlikely to be attributable to protein misfolding or interaction with Syx1. Next, a lipid mixing assay was performed to verify the mutation as previously described. Consistent with previous results, no fusion was detected between liposomes reconstituted with Munc18-1/Syx1 (t-liposomes) and liposomes containing Syb2 (v-liposomes) with the addition of SNAP-25, Synaptotagmin-1 (Syt1) cytoplasmic domain C2AB and Ca^2+^ but lacking Munc13-1. Further addition of the Munc13-1 C1C2BMUN domain (M13) promoted SNARE-mediated fusion between v-liposomes and t-liposomes (Fig. 1E, F). Interestingly, the TMR mutation was unable to promote liposome fusion despite C1C2BMUN addition (Fig. 1E, F). Together, these in vitro results revealed a set of residues in the hinge loop that were functionally important for Munc18-1/Syx1 transition to the SNARE complex.

### The TMR mutation abolished synaptic vesicle exocytosis

**Fig. 2:**
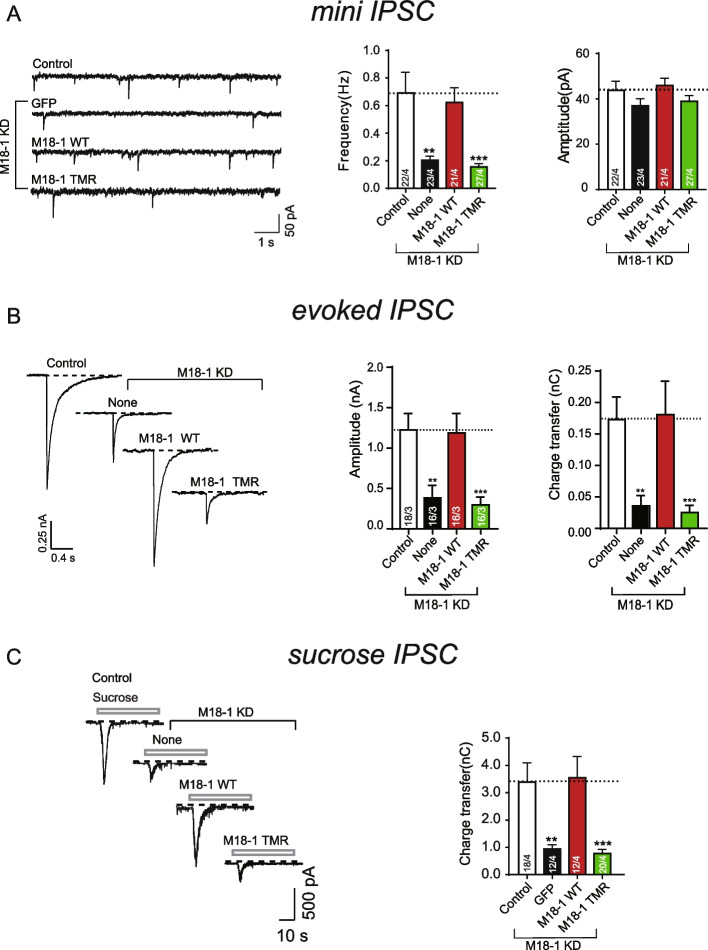
Residues T323/M324/R325 are crucial for synaptic vesicle exocytosis. **A** Sample traces (left), summary graphs of frequency (middle), and amplitude (right) of mIPSC. Data were recorded from cultured cortical neurons infected with control lentivirus (control), lentivirus expressing Munc18-1 shRNA (none), and rescued expressing sequences of Munc18-1 WT (WT) or Munc18-1 TMR mutant (TMR). **B** Sample traces (left), summary graphs of amplitude (middle), and charge transfer (right) of evoked IPSCs triggered by action potential recorded from cultured cortical neurons as described in **A**. **C** Sample traces (left) and summary graphs of charge transfer (right) of hypertonic sucrose-evoked IPSC recorded from cultured cortical neurons as described in **A**. Data are presented as mean ± SEM. Statistical significance was analyzed by Student’s *t* test; ^**^, *P* < 0.01; ^***^, *P* < 0.001. Cells recorded are from at least three independent cultures, and the cell numbers are shown in bars. KD, knockdown

Considering that Munc18-1 plays a pivotal role in regulating neurotransmitter release and that several studies have revealed that mutations in Munc18-1 domain 3a have a significant effect on synaptic vesicle exocytosis [18, 29, 49, 50], we carried out a knockdown-rescue experiment to test the effect of TMR mutation on synaptic vesicle exocytosis in cultured mouse cortical neurons. The expression of endogenous Munc18-1 was suppressed by a designed short hairpin RNA (shRNA) [29, 51]. Next, we monitored synaptic exocytosis when we rescued the expression of exogenous WT Munc18-1 and TMR. Consistent with previous results, knockdown (KD) of endogenous Munc18-1 strikingly impaired the frequency of spontaneous mIPSCs (mini-inhibitory postsynaptic current) and the amplitude and charge transfer of action potential-evoked IPSCs (Fig. 2A, B). However, expression of WT Munc18-1 rather than the TMR mutant rescued both mIPSCs and evoked IPSCs (Fig. 2A, B). In addition, the amplitudes of mIPSCs were not altered under any condition, suggesting that Munc18-1 knockdown mainly exerts presynaptic effects (Fig. 2A).

In addition, the Munc18-1 expression level was monitored by western blotting, and synapse formation and localization of Munc18-1 were measured by immunofluorescence. The results showed that both the Munc18-1 WT and the TMR mutant were steadily expressed in cultured neurons (Additional file 1: Fig. S3 A, B), were targeted to synapses (Additional file 1: Fig. S4 A, B), and supported synaptic formation without influencing the number and size of synapses (Additional file 1: Fig. S4 C, D).

Furthermore, we tested the functional defect of TMR mutation in vesicle priming by measuring the size of the readily releasable pool (RRP) of vesicles induced by hypertonic sucrose solution [52]. The charge transfer of sucrose-induced IPSCs was strongly reduced by Munc18-1 deficiency and rescued by the expression of WT Munc18-1 (Fig. 2C). Moreover, the TMR mutation exhibited a similar inability to rescue the RRP (Fig. 2C), indicating that residues T323/M324/R325 in the hinge loop are crucial for vesicle priming. Together, these in vitro and in vivo results suggest that the TMR mutation impairs synaptic priming due to impaired SNARE complex assembly and membrane fusion initiated by Munc18-1/Syx1.

### The TMR mutation remained Syx1 transport unchanged

Several studies have revealed that Munc18-1 plays an essential role in the chaperone and localization of Syx1 at the plasma membrane, which depends on the proper interaction between Munc18-1 and Syx1 [24, 51]. Impairing the interaction will perturb the localization of Munc18-1 and Syx1 at the plasma membrane and thus influence vesicle docking and priming. The results of size-exclusion chromatography and GST pull-down experiments indicate that the TMR mutant retains the ability to interact with Syx1 (Additional file 1: Fig. S2 and Fig. 1D). Nonetheless, to substantiate the binding results in vivo, we detected the distribution of Munc18-1 and Syx1 in HEK293T cells through confocal imaging experiments. Munc18-1 and Syx1 were expressed and fused with the fluorescent proteins EGFP and mCherry2, respectively.

**Fig. 3:**
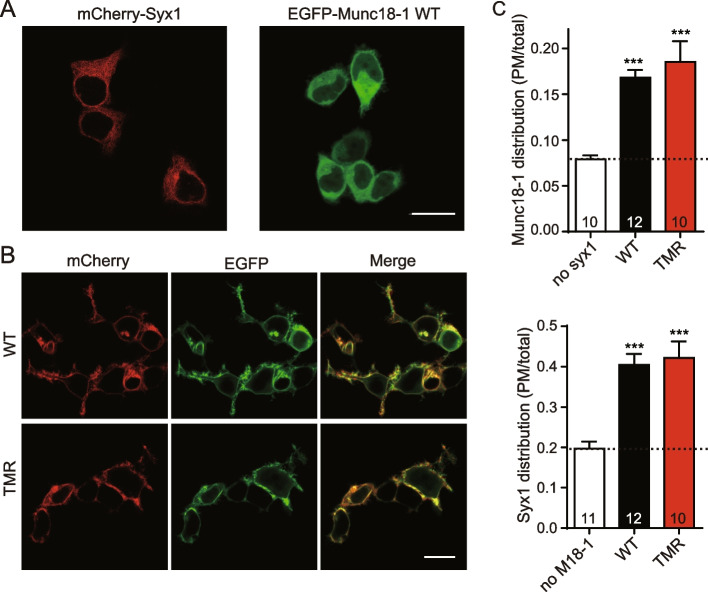
The TMR mutation supports Syx1 transport. **A** Representative images of HEK293T cells transfected with plasmids expressing EGFP-Munc18-1 and mCherry2-Syx1 respectively. **B** Representative images of HEK293T cells cotransfected with mCherry2-Syx1 and EGFP-Munc18-1 WT (WT) or TMR mutant (TMR). **C** Quantification of Munc18-1 and Syx1 distribution on the plasma membrane. Pictures used for analysis are from three independent cultures, and the numbers are listed in the bars. Data are shown as mean values ± SEM. Statistical significance was analyzed by Student’s *t* test. ^***^, *P* < 0.001. Scale bar: 20 μm; PM: plasma membrane

We found that Munc18-1 and Syx1 were not widely distributed on the cell membrane when expressed separately but mainly accumulated around the perinuclear region (Fig. 3A). Next, coexpression of WT Munc18-1 and Syx1 enabled the majority of these two proteins to localize on the cell membrane, with only a small amount of fluorescence in intracellular compartments (Fig. 3B, C). Similarly, the TMR mutant and Syx1 were properly distributed at the plasma membrane when coexpressed (Fig. 3B, C). Overall, we inferred that the priming deficiency of TMR mutation was not caused by Syx1 transport but selectively arose from SNARE assembly failure.

### The TMR mutation preserved the interaction between isolated Munc18-1 and Syb2

**Fig. 4:**
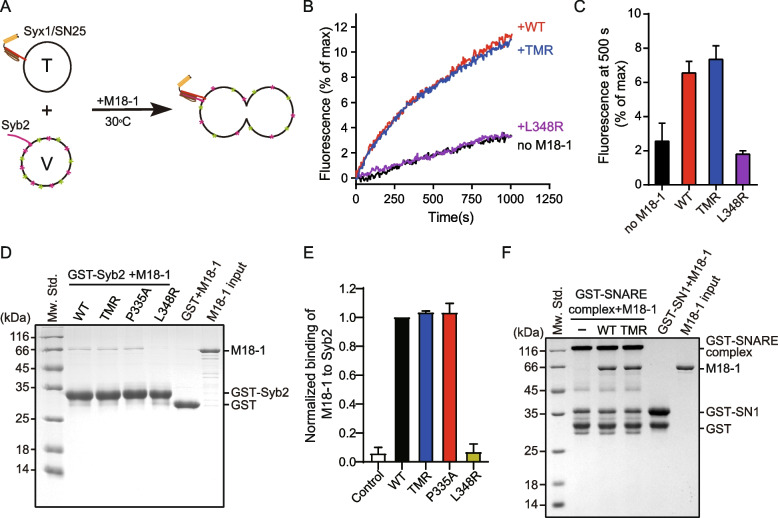
The TMR mutation supported Syb2 binding to isolated Munc18-1.** A** Illustration of Munc18-1-promoted reconstituted liposome fusion between v-liposomes bearing Syb2 (1–116) and t-liposomes containing Syx1 (1–288)/SNAP-25. V-liposomes were prepared by including NBD-PE and Rhodamine-PE, and NBD fluorescence at 538 nm was monitored. **B** Traces of lipid mixing reactions promoted by Munc18-1. Traces represented are from one of three independent replicates. All reactions were performed at 30 °C. **C** Quantification of the results shown in **B**. Data are presented as mean ± SD, *n* = 3. **D** Interaction between Munc18-1 and Syb2 detected by GST pull-down assay. The representative gel shown was from one of three replicates. **E** Quantification of integrated densities of Munc18-1 bands. The band densityy of WT Munc18-1 was taken as 1. Data are presented as mean values ± SD, *n* = 3. **F** Effect of TMR mutation on the Munc18-1 binding SNARE complex detected by GST pull-down assay. The SNARE complex was assembled by incubating GST-SN1, SN3, the cytoplasmic domain of Syb2 (29–93) and Syx1 (2–253) overnight at 4 °C. Bands of Munc18-1 and the SNARE complex are indicated in the figure. Representative gel displayed is from one of three independent replicates

Munc18-1 functions as a template to organize SNARE motifs to assemble the SNARE complex efficiently, and the interaction between Munc18-1 and Syb2 has attracted extensive attention [26, 27, 30, 41]. Based on the crystal structure of Vps33/Nyv1 and cryo-EM structure of Munc18-1/Syx1/Syb2 [28, 30], residues T323, M324, and R325 are located at the bottom of the extended domain 3a hinge loop and likely contribute to binding Syb2. We next determined whether the priming deficiency of the TMR mutation was triggered by the impaired interaction between Munc18-1 and Syb2. We first detected the stimulatory function of Munc18-1 in fusion between v-liposomes containing Syb2 and t-liposomes reconstituted with the t-SNARE complex (Syx1/SNAP-25) (Fig. 4A). The experiment selectively focused on the stimulatory function of Munc18-1, which depends on the interaction between Munc18-1 and v-SNARE and t-SNARE [53, 54]. Consistent with previous results, fusion between v-liposomes and t-liposomes exhibited a moderate rate, and further addition of Munc18-1 WT and the TMR mutant greatly accelerated the reaction (Fig. 4B, C). In contrast, the L348R mutation failed to promote the fusion (Fig. 4B, C) due to the impairment of binding Syb2. The Munc18-1-promoted lipid mixing results suggest that the TMR mutation does not affect Syb2 binding to the isolated Munc18-1. To corroborate this notion, we next detected the interaction between Munc18-1 and Syb2 directly by GST pull-down experiments. As shown by SDS–PAGE, Munc18-1 WT exhibited similar binding affinity to Munc18-1 bearing the P335A mutation that can extend the domain 3a hinge loop (Fig. 4D, E), which was consistent with previous results [41], indicating that domain 3a tends to adopt an extended conformation when Munc18-1 no longer binds Syx1. As expected, the TMR mutant also supported the interaction, similar to WT Munc18-1 (Fig. 4D, E), which was consistent with the Munc18-1-promoted lipid mixing results. In contrast, the L348R mutation completely abolished the interaction (Fig. 4D, E), which was in line with previous reports [27, 41]. These results together indicate that the TMR mutation has no effect on the interaction of isolated Munc18-1 with Syb2.

Several studies have proposed that domain 3a is responsible for binding the SNARE complex directly. The loss-of-function mutation KE/5I impairs Munc18-1 binding to the SNARE complex, and the gain-of-function mutation P335A, which extends domain 3a, enhances the binding affinity [35, 45]. We next carried out GST pull-down assay to test the interaction. The SNARE complex was prepared by incubating GST-SN1 (residues 11–82 of SNAP-25), SN3 (residues 141–203 of SNAP-25), Syx1, and Syb2, and then Munc18-1 WT and TMR mutants were added into the SNARE complex. As shown by SDS–PAGE, the Munc18-1 TMR mutation supported Munc18-1 binding to the SNARE complex, which was similar to the binding by Munc18-1 WT (Fig. 4F). These results exclude the possibility that TMR mutation affects the process after SNAREs are fully assembled.

### The TMR mutation impaired the extension of the domain 3a hinge loop

At least two conformational changes are involved in the transition from Munc18-1/Syx1 to the SNARE complex, including the opening of the Syx1 linker region and the extension of the Munc18-1 domain 3a hinge loop [25, 27, 41]. In addition, we have revealed that the opening of the Syx1 linker region is catalyzed by the MUN domain, which depends on the direct interaction between Munc18-1/Syx1 and the MUN domain. Opening of the linker region subsequently leads to the extension of domain 3a and enables Syb2 to bind domain 3a [25, 29, 42]. Next, we carried out smFRET experiment and GST pull-down assay to detect the effect of TMR mutation on two conformational changes.

**Fig. 5:**
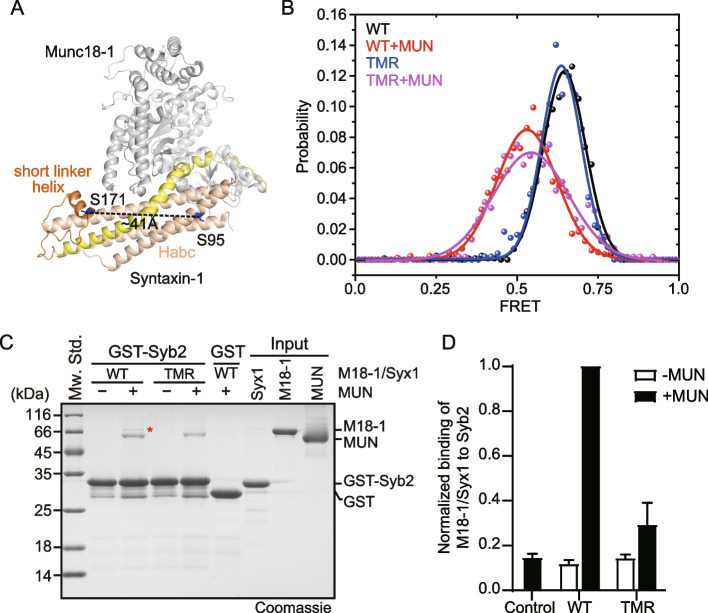
Crucial role of residues T323/M324/R325 in the extension of domain 3a hinge loop.** A** Position of FRET pair labeled on Syx1 to monitor the relative movement between the linker region and Habc in the crystal structure of Munc18-1/Syx1 (PDB ID: 3C98). **A** Syx1 S95C/C145S/S171C mutation was generated for stochastic labeling of the FRET pair Alexa-555 and Alexa-647. **B** Representative histograms of smFRET efficiency of FRET-pairs labeled Syx1 binding Munc18-1 WT or TMR mutant in the absence and presence of MUN domain. Munc18-1/Syx1 was fixed on a coverslip modified with PEG/PEG-NTA-Co^2+^ via the His tag at the C-terminus of Syx1. **C** TMR mutation impaired Syb2 binding to the Munc18-1/Syx1 complex in the presence of the MUN domain, as detected by GST pull-down. Bands of Munc18-1 were highlighted with red asterisk in Coomassie brilliant blue stained SDS–PAGE. Representative gel displayed is from one of three independent replicates. **D** Quantification of the integrated densities of the Munc18-1 bands in **C**. The band density of WT Munc18-1 in the presence of the MUN domain was taken as 1. Data are presented as mean values ± SD, *n* = 3

To detect the conformational change in the linker region, Syx1 was stochastically dually labeled with Alexa-555 and Alexa-647 at the positions of serine 95 (on the rigid Habc domain) and serine 171 (on the flexible linker region), and the two serines were mutated to cysteines for convenient labeling (Fig. 5A). Variation in the distance between two regions was reflected by the changes in FRET efficiency between two fluorescent molecules that were monitored by using total internal reflection fluorescence microscopy. Consistent with previous observations, dual-labeled Syx1 exhibited high FRET efficiency when bound to WT Munc18-1, in accordance with the closed conformation (Fig. 5B). Furthermore, the addition of the MUN domain shifted the FRET efficiency population to a lower location (Fig. 5B), reinforcing the notion that the MUN domain induces a “closed-to-open” change in the Syx1 linker region. When bound to the TMR mutant, the FRET efficiency of Syx1 exhibited a similar effect to that when bound to WT Munc18-1, regardless of the absence or presence of the MUN domain (Fig. 5B), indicating that the TMR mutation does not affect the closed conformation formation and the “closed-to-open” transition of the Syx1 linker region in the Munc18-1/Syx1 complex.

The extension of the domain 3a hinge loop is inconvenient to detect directly, but several studies have revealed that Syb2 binds to domain 3a in an extended conformation [29, 34, 41]. Thus, we utilized a previously established GST pull-down assay combined with western blotting to detect the interaction of Munc18-1/Syx1 with Syb2 in the presence of the MUN domain, in which Syb2 binds to the extended domain 3a driven by MUN opening the Syx1 linker region. Nearly no Munc18-1/Syx1 WT (reflected by bands of Munc18-1) was found to bind GST-Syb2 in the absence of the MUN domain, which was equivalent to the GST control (Fig. 5C, D). Further addition of the MUN domain enhanced the binding of WT Munc18-1/Syx1 (Fig. 5C, D). However, Munc18-1/Syx1 bearing the TMR mutation showed similar binding affinity to the GST control in the presence and absence of the MUN domain (Fig. 5C, D). The binding of Munc18-1/Syx1 to Syb2 was further confirmed by western blotting (Additional file 1: Fig. S5). After comprehensive consideration of the performance of TMR mutation in the opening of the linker region and interaction with isolated Munc18-1, we concluded that TMR mutation selectively hindered the extension of domain 3a hinge loop, causing the Syb2-binding surface to be hidden. These results together indicate that residues T323, M324, and R325 are crucial for the extension of domain 3a hinge loop.

## Discussion

In the model of the Munc13-catalyzed transition from the Munc18-1/Syx1 complex to the SNARE complex, the closed conformation in the Syx1 linker region and the bent conformation in Munc18-1 domain 3a provide suitable energy barriers to hinder the SNARE complex assembly and realize master regulation via rapid conformational changes [21, 22, 27, 31, 55]. Following the opening of the Syx1 linker region catalyzed by the Munc13-1 MUN domain, the extension of the domain 3a hinge loop provides a template for orchestrating SNARE nucleation [29, 42]. Based on these previous reports, it is concluded that Munc18-1 domain 3a regulates SNARE assembly and synaptic exocytosis through a variety of mechanisms: (i) together with domain 1, locking Syx1 into a closed conformation while domain 3a adopts a furled conformation [21, 23, 56]; (ii) providing an MUN interaction site to mediate the MUN-catalyzed opening of the Syx1 linker region [29]; and (iii) acting as a template to bind the SNARE motifs of Syb2 and Syx1 when the hinge loop is unfurled [26, 27, 29, 30]. The mechanism of conformational change in the Syx1 linker region has attracted extensive attention. However, the regulatory mechanism of conformational change in domain 3a remains unclear. In this study, by the combined utilization of experimental methods in vitro and in vivo, we identified three residues (T323/M324/R325) in the domain 3a hinge loop (Fig. 1A) that are crucial for the activity of Munc18-1 in SNARE complex assembly (Fig. 1B, C) and synaptic exocytosis (Fig. 2), and elucidated the importance of the three amino acids for the extension of domain 3a.

The previously reported residue D326 is likely to form hydrogen bonds with surrounding amino acids, thus putting domain 3a into an autoinhibitory state and establishing an energy barrier to SNARE assembly [27]. We further characterized the role of amino acid residues around D326 in the conformational change of domain 3a. T323A, M324A, and R325A were identified for various degrees of inactivation in the transition from Munc18-1/Syx1 to the SNARE complex (Additional file 1: Fig. S1). As expected, the ternary mutation T323A/M324A/R325A showed a stronger negative effect on SNARE complex assembly (Fig. 1B, C) and disrupted SNARE-mediated liposome fusion starting from Munc18-1/Syx1 (Fig. 1E, F). Moreover, the ternary mutation completely hindered vesicle priming and fusion by the electrophysiology technique (Fig. 2), which is consistent with the in vitro results and suggests residues T323, M324, and R325 are important for Munc18-1 to promote SNARE complex assembly. Similar to other single-residue mutations in Munc18-1 domain 3a (except for the P335A mutation), the TMR mutation had no effect on the Syx1 interaction and transportation to the plasma membrane (Figs. 1D and 3).

The molecular mechanism by which domain 3a regulates SNARE complex assembly and the priming of synaptic vesicles has attracted much attention. Regarding whether the loss-of-function TMR mutation shared a similar mechanism to the mutations reported previously, the first notion to come to mind was the impaired Munc18-1-Syb2 binding. Based on the crystal structure of Vps33/Nyv1 and cryo-EM structure of Munc18-1/Syx1/Syb2 [28, 30], residues T323/M324/R325 that locate at the bottom of the Syb2-binding groove constituted by Munc18-1 helix11 and helix12 likely contribute to binding the N-terminal region of Syb2. We first used the previously established Munc18-1-stimulated lipid mixing assay to illustrate the interaction between Munc18-1 and Syb2 [53, 54]. The t-SNARE complexes were reconstituted to liposomes to selectively focus on the stimulatory function of Munc18-1 during the priming process, which is independent of the inhibitory function. The stimulating function depends on the interaction of Munc18-1 with t- and v-SNAREs [53, 54]. Consistent with previous results, the L348R mutation designed to disrupt Syb2 binding [41] failed to promote fusion (Fig. 4B, C). Interestingly, the TMR mutation preserved the Munc18-1 fusogenic activity (Fig. 4B, C), indicating that residues T323/M324/R325 are likely not responsible for binding with the N-terminal region of Syb2. We speculated that a certain degree of freedom for the N-terminus of the SNARE motif is favorable for nucleation. However, in the presence of NSF/α-SNAP in vivo, the Syx1/SNAP-25 complex is dissociated by Munc18-1 and converts to the Munc18-1/Syx1 pathway [31]. The binding of Munc18-1 to GST-Syb2 was difficult to identify by GST pull-down experiments when all samples were analyzed with SDS–PAGE after extensive washing, as Munc18-1 bound to the medium nonspecifically. Alternatively, the application of reduced glutathione could prevent the problem with no need for a high concentration of Munc18-1. Interestingly, Munc18-1 WT and TMR mutant exhibit similar Syb2 binding affinity to the P335A mutant that was designed to extend the domain 3a hinge loop [41]. Combined with the crystal structure of Munc18-1 KKEE, Munc18-1/Syx4 N-peptide, squid sec1, and Munc18-2, in which domain 3a preferentially adopts an extended conformation and has a certain flexibility [29, 46, 57, 58], it can be concluded that the TMR mutation might not change the extension tendency and flexibility of Munc18-1 domain 3a when it no longer binds Syx1.

Under physiological conditions, Munc18-1 clamps tightly to Syx1 and chaperons it to the plasma membrane. In the Munc18-1/Syx1 complex, the domain 3a hinge loop is furled, that is, incompatible for Syb2 binding. The MUN domain interacts with Munc18-1/Syx1 to induce the opening of the Syx1 linker region and extension of domain 3a, enabling Syb2 to bind the extended domain 3a [25, 29, 34]. Multiple cysteines in Munc18-1 render it impractical to detect the conformational change of the domain 3a by smFRET; thus, we can characterize the extension of domain 3a via Syb2 binding to domain 3a in Munc18-1/Syx1[29]. Interestingly, smFRET experiments revealed that the TMR mutation supports the MUN domain opening the Syx1 linker region (Fig. 5B) but leads to no interaction between Syb2 and Munc18-1/Syx1 despite the addition of the MUN domain (Fig. 5C, D). These data suggest that residues T323/M324/R325 selectively hinder the extension of the domain 3a hinge loop but have no effect on Syb2 binding to isolated Munc18-1 and the interaction between Munc13-1 and Munc18-1.

During the transition from Munc18-1/Syx1 to the SNARE complex, a series of processes are realized by weak interactions among proteins and conformational changes. The illustration of the relationship between the opening of the Syx1 linker region and the extension of the domain 3a hinge loop lays a foundation for exploring the functional mechanism of domain 3a. According to our previous work, both the extension of Munc18-1 domain 3a and the opening of the Syx1 linker region require MUN to bind Munc18-1/Syx1. Considering that MUN does not display striking promotion of SNARE assembly starting with Munc18-1/Syx1 LEAA, but the extension of domain 3a has no effect on the closed conformation of the Syx1 linker region in the Munc18-1 (P335A)/Syx1 complex [29], we speculated that the LEAA mutation allowed Munc18-1/Syx1 to completely bypass the need for the MUN domain, leading to both the extension of domain 3a and the opening of the Syx1 linker region. In other words, the opening of the Syx1 linker region driven by the MUN domain is an early event that leads to the extension of the domain 3a hinge loop, but not vice versa. This conclusion is in accordance with the cryo-EM structure of Syb2/Munc18-1/Syx1, which represents a crucial intermediate of SNARE assembly, and the crystal structure of Vps45/Tlg2. In two structures, the linker regions of Tlg2 and Syx1 adopt a constitutively open conformation, and the N-terminus of the SNARE motif contacts the extended domain 3a of Vps45 or Munc18-1 [28, 59]. Single-molecule optical tweezers provide an efficient approach to identify the different states, energetics, and kinetics involved in SNARE assembly chaperoned by Munc18-1 [26, 60–62]. These findings also revealed that Munc18-1 clamps the open Syx1 to form the template complex with domain 3a extension [26]. These structural and experimental results together strengthen the notion that domain 3a adopts an extended conformation once the Syx1 linker region adopts an open conformation. Recent studies revealed that deletion of the disordered region (residues 314–326) of domain 3a, replacement with a glycine- and serine-rich sequence, or deletion of residues 324–339 impaired the template intermediate (Munc18-1/Syx1/Syb2) formation and SNARE complex assembly [26, 62]. These results reinforce that the domain 3a hinge loop region is crucial for the template function of Munc18-1. Taken together, our results further reveal the pivotal role of Munc18-1 domain 3a in SNARE complex assembly and synaptic exocytosis, and elucidate the key residues for domain 3a to assume different conformations during SNARE complex assembly.

## Conclusions

At least two conformational changes, including the “closed-to-open” transition in the Syx1 linker region and the “bent-to-extended” transition in the Munc18-1 domain 3a loop region during the transition from Munc18-1/Syx1 to the SNARE complex, provide key points for master regulation by other components. Munc18-1 extends its domain 3a and functions as a template to bind Syb2 and Syx1 simultaneously for exquisite organization of SNARE complex assembly. Here, we identified the essential roles of residues T323, M324, and R325 in the extension of domain 3a during the transition from Munc18-1/Syx1 to the SNARE complex. Mutation of these residues caused severe defects in synaptic exocytosis in vivo and in SNARE complex assembly in vitro. Together with previous results, our data further support the model that Munc18-1 functions as a template to bind Syb2, requiring the conformational extension of domain 3a in Munc18-1/Syx1, and particular residues in the hinge loop enable rapid release of the autoinhibitory conformation of domain 3a after the opening of the Syx1 linker region.

## Methods

### Plasmid construction

The full-length Munc18-1 (residues 1–594) and Syb2 (1–116) and cytoplasmic fragments of rat Syx1 (residues 2–253) and Syb2 (residues 29–93) were cloned into the pGEX-6P-1 vector. Fragments of human SNAP-25 SN1 (residues 11–82) and SN3 (residues 141–203), rat Syt1 C2AB domain (residues 140–421), and Munc13-1 MUN domain (residues 933–1,407, EF, 1,453–1,531) were cloned into the pGEX-KG vector. Full-length SNAP-25 (with its four cysteines mutated to serines), Syx1 (2–253, with His tag at C-terminus) WT, and mutant (S95C/C145S/S171C) were cloned into the pET28a vector. Munc18-1/Syx1 (residues 2–253) and the t-SNARE complex Syx1 (183–288)/SNAP-25 were constructed into the pETDuet-1 vector (Novagen). All mutations were generated by QuickChange Site-Directed Mutagenesis Kit (Stratagene). For confocal imaging, the full-length sequences of Munc18-1 and Syx1 were subcloned into the vectors pEGFP-C1 (Clontech) and mCherry2-C1 (Addgene), respectively. The Munc13-1 C1C2BMUN fragment (residues 529–1,407, EF, 1,453–1,531) was cloned into the pFastBac™ HT B vector and expressed in insect cells (Sf9) as described previously [31].

### Recombinant protein expression and purification

All recombinant proteins were expressed in *Escherichia coli* BL21 (DE3) except for the Munc13-1 C1C2BMUN fragment. Cells were grown at 37 °C for 3 h with a cell density of OD_600_≈ 0.8 and induced with 0.5 mM IPTG (isopropyl-β-thiogalactoside) at 16, 25, or 37 °C for the expression of various proteins. Cells were centrifuged at 4000 rpm at 4 °C for 20 min. For GST-fused proteins, cells were resuspended in PBS with the addition of 1 mM PMSF, 5 mM DTT (DL-dithiothreitol), and 1 mM EDTA (pH 8.0), lysed with a high-pressure crusher, and centrifuged at 16,000 rpm at 4 °C for 30 min. Supernatants were collected and incubated with 1 ml of glutathione Sepharose 4B (GE Healthcare) affinity media at 4 °C for 2 h. Thrombin (Sigma) or PreScission protease were used to remove the GST tag at the N-terminal of proteins. GST-tagged proteins were eluted by buffer containing 20 mM GSH (pH 8.5) for the GST pull-down assay. For 6 × His-fused proteins, cells were resuspended in Tris–HCl buffer (50 mM Tris–HCl pH 8.0, 150 mM NaCl, 10% glycerol) with the addition of 5 mM imidazole. Bacterial suspensions were lysed and centrifuged as described above. Supernatants were mixed with Ni–NTA agarose (Qiagen) affinity media at 4 °C for 2 h. After an extensive wash to reduce nonspecific binding of proteins, His-tagged proteins were eluted by Tris–HCl buffer containing 250 mM imidazole. All proteins were loaded onto Superdex 200 pg or Superdex 75 pg size-exclusion chromatography columns (GE Healthcare) for further purification to remove other contaminants and aggregates.

### Native PAGE assay

Purified Munc18-1 and Syx1 were incubated at 4 °C overnight at concentrations of 6 and 5 μM, respectively, to form Munc18-1/Syx1 extensively. To detect the transition from Munc18-1/Syx1 to the SNARE complex, 10 μM Syb2 and 10 μM SNAP-25 were mixed with Munc18-1/Syx1 (final concentration of 3 μM/2.5 μM) in the absence or presence of 30 μM MUN domain in a final volume of 10 μl and incubated at 30 °C for 1.5 h. The mixture was loaded into the nondenaturing gel, running at 4 °C for 2 h. The nondenaturing gel was prepared with 15% polyacrylamide in the separating gel (pH 8.4) and 5% polyacrylamide in the stacking gel (pH 6.8) free of SDS (sodium dodecyl sulfate). The gel was stained with Coomassie Brilliant Blue and analyzed by ImageJ (NIH) with quantification of the density of each Munc18-1/Syx1 band. Each reaction was repeated at least 3 times.

### Proteoliposome reconstitution and fusion assay

All lipids (purchased from Avanti Polar Lipids) were dissolved and stored as previously described. The lipid mixture was dried with nitrogen and placed under vacuum for 3 h to remove the organic solvents extensively. Lipids were resuspended in buffer H (25 mM HEPES pH 7.4, 150 mM NaCl, 10% glycerol) containing 0.2 mM TCEP (Sigma–Aldrich) and 1% (w/v) CHAPS (Amresco). Purified proteins were added to suspended lipids (with a lipid final concentration of 5 mM), incubated on ice for 1 h, and then dialyzed three times against buffer H supplied with 1 mM DTT at 4 °C. One gram/liter Bio-Beads SM2 (Bio-Rad) were added into the last dialysis buffer to remove the detergent extensively.

To monitor liposome fusion starting from Munc18-1/Syx1, liposomes reconstituted with Munc18-1/Syx1 (1–288) (t-liposomes) contained 58% POPC, 15% POPE, 20% DOPS, 2% PIP2, and 5% DAG, and liposomes bearing Syb2 (1–116) (v-liposomes) contained 60% POPC, 17% POPE, 20% DOPS, 1.5% NBD-PE, and 1.5% Rhodamine-PE. The protein-to-lipid ratio was 1:500 for t-liposomes and 1:200 for v-liposomes. Liposome-Syb2 (0.25 mM lipids) and liposome-Munc18-1/Syx1 (0.25 mM lipids) were mixed in the presence of 5 μM SNAP-25, 2 μM Syt1 C2AB fragment, 1 μM Munc13-1 C1C2BMUN fragment, and 1 mM CaCl_2_. To detect liposome fusion beginning with the t-SNARE complex (Syx1/SNAP-25), t-liposomes reconstituted with Syx1 (1–288)/SNAP-25 were prepared with 60% POPC, 20% POPE, 10% DOPS, and 10% cholesterol, and v-liposomes bearing Syb2 (1–116) were prepared with 60% POPC, 17% POPE, 10% DOPS, 10% cholesterol, 1.5% NBD-PE, and 1.5% Rhodamine-PE. The protein-to-lipid ratio was 1:500 for t-liposomes and 1:200 for v-liposomes. Fusion reactions were performed as previously reported [53, 54].

All lipid mixing reactions were performed using a QM-40 spectrophotometer excited by wavelength 460 nm at 30 °C. CHAPS (1%, w/v) was added to terminate the reactions. NBD emission fluorescence at 538 nm was monitored. Fluorescence intensity was calculated with the following formula:* E* = (*F*_obs_ − *F*_0_)/(*F*_max_ − *F*_0_) × 100% (*F*_obs_, the observed fluorescent intensity; *F*_0_: the initial fluorescent intensity; *F*_max_, fluorescent intensity upon addition of 1% CHAPS). Each reaction was repeated at least three times.

### GST pull-down assay

To monitor the interaction between Munc18-1 and Syx1, 5 μM N-terminal GST-tagged Syx1 (2–253) was mixed with 10 μM Munc18-1 and 10 μl glutathione Sepharose 4B affinity media (GE Healthcare) and rotated at 4 °C for 2 h. To detect the interaction between isolated Munc18-1 and Syb2 directly, 3 μM GST-Syb2 and 6 μM Munc18-1 were incubated with 10 μl glutathione Sepharose 4B affinity media at 4 °C for 3 h in 100 μl of HEPES buffer with the addition of 0.05% Triton X-100 (v/v). Affinity media was washed 3 times with binding buffer, and all buffer was removed after the last washing. Thirty-five microliters of HEPES buffer containing 30 mM reduced glutathione was added to the affinity media to elute GST-tagged Syb2. To detect the binding between Munc18-1 and the SNARE complex, GST-SN1, SN3, Syb2 (29–93), and Syx1 at a ratio of 1:1.2:1.2:1 were preincubated overnight to form the SNARE complex. GST-tagged SNARE complex (2 μM) and Munc18-1 (5 μM) were incubated with 10 μl glutathione Sepharose 4B affinity media at 4 °C for 3 h. To detect the interaction between Munc18-1/Syx1 and Syb2 in the presence of the MUN domain, 10 μM Munc18-1 and 12 μM Syx1 were preincubated overnight to form the Munc18-1/Syx1 complex extensively. 6 × His tag at the C-terminal of Syx1 was used for detection by western blotting. GST-Syb2 (3 μM) and Munc18-1/Syx1 (5 μM) were incubated with 10 μl of glutathione Sepharose 4B affinity media at 4 °C for 4 h in the presence or absence of 10 μM MUN domain. To reduce nonspecific binding, all reactions were performed in 100 μl of HEPES buffer with the addition of 0.05% Triton X-100 (v/v). Affinity media was washed 3 times with binding buffer and analyzed by SDS–PAGE. For western blotting, Munc18-1 rabbit polyclonal antibody (#11,459–1-AP, purchased from Proteintech, Wuhan, China) was applied at a dilution of 1:5000, and mouse monoclonal anti-6 × His (Proteintech; 66,005–1-lg) was applied at a dilution of 1:2500 to reveal the binding of Munc18-1 and Syx1 respectively.

### Cell culture

HEK293T cells (CRL-11268, ATCC) were cultured with Dulbecco’s modified Eagle’s medium (Gibco) supplied with 10% (v/v) fetal bovine serum (Gibco) and penicillin–streptomycin (50–50 μg/ml) and placed in a 37 °C cell incubator (Thermo) supplied with 5% CO_2_. Cortical neurons were dissociated from newborn pups of Kunming mice (obtained from the Hubei Provincial Center for Disease Control and Prevention, Wuhan, Hubei) as described previously [25]. Briefly, the mouse cerebral cortex was dissected and digested with 0.25% trypsin–EDTA (Gibco) at 37 °C for 12 min and then plated on glass coverslips coated with poly-L-lysine (Sigma) at a density of 80,000 cells per 8 mm × 8 mm. Cells were cultured at 37 °C with 5% CO_2_ in MEM medium with the addition of 2% (v/v) B27 (Gibco), 0.5% (w/v) glucose (Sigma), 100 mg/l transferrin (Sigma), 5% (v/v) fetal bovine serum (Gibco), and 2 mM Ara-C (Sigma).

### Lentivirus preparation

Lentivirus preparation was carried out in HEK293T cells cotransfected with lentivirus vectors (L309) and three helper plasmids (pRSV-REV, pMDLg-pRRE, and pVSVG). Plasmids were transfected into HEK293T cells via PEI (polyethylenimine) with a molar ratio of L309:pRSV-REV:pMDLg-pRRE:pVSVG:PEI = 3:2:2:1:24. Four micrograms of plasmids and 12 μl of PEI were incubated in 100 μl of Opti-MEM (Gibco) medium at room temperature for 30 min. Then, the mixture was added to HEK293T cells and cultured for 48 h. Then, virus-containing medium was collected and centrifuged at 1000 × *g* for 3 min, and the supernatants were harvested. To concentrate the virus, the virus-containing medium acquired above was overlaid on a sucrose-containing buffer (50 mM Tris–Cl, pH 7.4, 100 mM NaCl, 0.5 mM EDTA) at a ratio of 4:1 (v/v). After centrifugation at 10,000 × *g* at 4 °C for 4 h, the precipitate was collected and resuspended in PBS by statically incubating overnight at 4 °C.

### Electrophysiological recording

A HEKA EPC10 amplifier (HEKA) was used for electrophysiological recordings in whole-cell patch clamping mode as described previously [63]. Patch pipettes were generated from borosilicate glass capillary tubes (World Precision Instruments, Inc.) by using a P-97 pipette puller (Sutter). Whole-cell pipette solution was prepared by 120 mM CsCl, 10 mM HEPES, 0.3 mM Na-GTP, 10 mM EGTA, and 3 mM Mg-ATP, and the pH was adjusted to 7.2 with CsOH; the cell bath solution was prepared by 140 mM NaCl, 5 mM KCl, 2 mM MgCl_2_, 2 mM CaCl_2_, 10 mM glucose, and 10 mM HEPES–NaOH, adjusted pH to 7.4 with NaOH. The osmotic pressure values of the pipette and cell bath solutions were adjusted to 305 and 315 with ddH_2_O, respectively. To isolate inhibitory postsynaptic currents (IPSCs), 20 μM 6-cyano-7-nitroquinoxaline-2,3-dione (CNQX) and 50 μM AP-50 were added to the bath solution. To monitor mini IPSCs, 1 μM tetrodotoxin (TTX) was added to the bath solution to block the action potential. The evoked IPSCs were recorded with 90 μA stimulus pulses. 0.5 M hypertonic sucrose was added to the bath solution to measure the size of the RRP. Neurons were electrophysiologically analyzed at DIV (days in vitro) 13–14.

### Confocal imaging

HEK293T cells were cultured in glass-bottom dishes (Nest) for 12 h and transfected with plasmid pEGFP-C1 expressing Munc18-1 and/or mCherry2-C1 expressing Syx1. Images were acquired by using a Nikon C2 confocal microscope equipped with a × 60 oil-immersion objective after cells were transfected for 24 h. Plasma membrane Syx1- or Munc18-1-specific fluorescence intensity divided by whole-cell total fluorescence intensity was calculated as the fluorescence distribution. Image detection and segmentation were achieved by using MATLAB (version 2010b) scripts written in-house.

### Immunostaining

Mouse cortical neurons infected with different lentiviruses were fixed in 4% paraformaldehyde and permeabilized with 0.2% Triton X-100, incubated with anti-Munc18-1 (mouse monoclonal antibody, Proteintech #67,137–1-Ig, at a dilution of 1:500) and anti-synapsin1 (rabbit polyclonal, Proteintech # 20,258–1-AP, at a dilution of 1:800) primary antibodies in PBS with 5% BSA, washed, and visualized using Alexa Fluor-488 goat anti-mouse (A11029) and Alexa Fluor-546 goat anti-rabbit (A11035) secondary antibodies (Molecular Probes) applied at a dilution of 1:500. Primary anti-synapsin1 antibodies were used to mark synapses. Images were acquired by using a Nikon C2 confocal microscope equipped with a × 60 oil-immersion objective. Identical settings were applied to all samples in each experiment. As in our previous report, we measured the average pixel intensities by manually tracing each dendrite, with a > twofold background signal [64]. We measured the Munc18-1-specific fluorescence intensity at synaptic locations that had been labeled by synapsin1 (Fsynapse) and at locations that were randomly picked elsewhere that were not colocalized with synapsin1 locations (Fnon-synapse). Δ*F* = *F*synapse–*F*non-synapse was calculated to determine the subcellular location of Munc18-1.

### Western blot

The expression of Munc18-1 and the TMR mutant in neurons at DIV 13–14 was quantitated by western blotting using Munc18-1 antibody (mouse monoclonal, Proteintech #67,137–1-Ig) at a dilution of 1:5000 and Beta Actin antibody (mouse monoclonal, Proteintech #66,009–1-Ig) at a dilution of 1:20,000. The integrated densities of each band were analyzed by ImageJ (NIH).

### Single-molecule FRET experiment

Detailed procedures for imaging chamber preparation and data collection were described previously [25, 29]. Syx1 (S95C/C145S/S171C) was incubated with Alexa Fluor 555 C2 maleimide (Invitrogen) and Alexa Fluor 647 C2 maleimide (Invitrogen) simultaneously at a ratio of 1:5:5. Samples in brown Eppendorf tubes were wrapped with tin foil to avoid light and gently rotated overnight at 4 °C. Excess dyes were removed using a PD-10 desalting column. One micromolar fluorescently labeled Syx1 and 2 μM Munc18-1 were incubated overnight at 4 °C to form Munc18-1/Syx1. The mixture was loaded into the imaging chamber at a final concentration of 0.5 μM and immobilized on the inner face of the imaging chamber through the 6 × His tag at the C-terminal of Syx1 at room temperature for 30 min. Unbound proteins were washed out with imaging buffer (25 mM HEPES-K^+^, 150 mM KCl, supplied with oxygen scavenger (20 units/ml glucose oxidase (from *Aspergillus niger*, Sigma–Aldrich), 1000 units/ml catalase (from bovine liver, Sigma–Aldrich), and 1% (w/v) glucose)). The mixture containing 30 μM MUN domain was injected into the imaging chamber and placed in the dark at 30 °C for 1 h.

## Supplementary Information

**Additional file 1:** **Fig. S1. **Mutations T323A, M324A and R325A impaired the MUN-catalyzed transition from Munc18-1/Syx1 to the SNARE complex. Representative native gel (upper) shown was from one of three replicates. Quantification of the integrated densities of Munc18-1/Syx1 bands is shown below the native gel. Data are presented as mean values ± SD, *n* = 3. **Fig. S2. **Peak elution volume analysis of Munc18-1 WT and mutants in the presence and absence of Syx1. All analyses were performed by size-exclusion chromatography on a Superdex 200 increase 10/300 GL column. **Fig. S3. **Expression of Munc18-1 TMR in cultured cortical neurons. (A) Quantification of Munc18-1 expression by western blot in neurons infected with control lentivirus (Control) or lentiviruses expressing Munc18-1 shRNA alone (None) or together with wild-type Munc18-1 (WT) or Munc18-1 TMR mutants. Representative results displayed are from one of three independent replicates. (B) Quantitative analysis of the integrated densities of Munc18-1/β-actin bands. The mean values ± SD are shown, *n* = 3. **Fig. S4. **The Munc18-1 TMR mutant does not alter synapse formation or the localization of Munc18-1 targeting to synapses in cultured cortical neurons. (A) Cultured cortical neurons infected with control lentivirus or with lentivirus expressing the Munc18-1 shRNA only (None), or together with wild-type Munc18-1 (WT) or the TMR mutant. Neurons were fixed and labeled by double immunofluorescence using Synapsin-1 (to mark synapses) and Munc18-1 antibodies along with fluorescently labeled secondary antibodies (see Materials and Methods). (A) Summary graphs of Synapsin-1-specific fluorescence intensities for all conditions as described above. (B) Summary graphs of Δ*F* (*F*_synapse_− *F*_non-synapse_) of Munc18-1-specific fluorescence intensity for all conditions as described above. Summary graphs of synapse number (C) and size (D), which were quantified from Synapsin-1-specific puncta. Data are presented as mean values ± SEM. Neurons analyzed are from three independent cultures. Numbers of cells/independent cultures analyzed are listed in the bars. Statistical assessments were performed by Student’s *t*-test comparing each condition to the indicated control experiment (****, *P*< 0.0001). **Fig. S5. **Interaction between Munc18-1/Syx1 and Syb2 in the presence of the MUN domain analyzed by western blotting. Immunoblotting data are shown with Munc18-1 monoclonal antibody and His-Tag monoclonal antibody to exhibit the binding of Munc18-1 and Syx1, respectively. Bands of Munc18-1 degradation are indicated with asterisks. Degradation is common in Munc18-1 and has no effect on Syx1 binding. Representative results displayed are from one of three independent replicates.

**Additional file 2.** Uncropped images of gels and Western blots shown in this paper.

**Additional file 3.** The individual data values for Fig. 1C, F, 2A-C, 3C, 4C, E, 5B, D, S1, S2, S3B, S4A-D.

**Additional file 4.** Original images of confocal imaging for Fig. 3A and B.

## Data Availability

All data generated or analyzed during this study are included in this published article and its supplementary information files. Uncropped gels and blots are provided in Additional file 2. Original data of replicated experiments used for quantification can be found in Additional file 3. Original images of confocal imaging for Fig. 3 are provided in Additional file 4.

## Abbreviations

*Syx1*: Syntaxin-1
*SN25*: SNAP-25
*Syb2*: Synaptobrevin-2
*SM*: Sec1/Munc18-like
*M18-1*: Munc18-1
*CATCHR*: Complexes associated with tethering containing helical rods
*DCVs*: Dense core vesicles
*C. elegans*: *Caenorhabditis elegans*
*Syt1*: Synaptotagmin-1
*M13*: Munc13-1 C1C2BMUN domain
IPTG: Isopropyl-β-thiogalactoside
SDS: Sodium dodecyl sulfate
DTT: DL-dithiothreitol
PEI: Polyethylenimine
IPSCs: Inhibitory postsynaptic currents
CNQX: 6-Cyano-7-nitroquinoxaline-2,3-dione
TTX: Tetrodotoxin
DIV: Days in vitro
WT: Wild-type
TMR: T323A/M324A/R325A
shRNA: Short hairpin RNA
mIPSCs: Mini-inhibitory postsynaptic current
RRP: Readily releasable pool
