# Supplementary material for: Identification of residues critical for the extension of Munc18-1 domain 3a
Source: BMC Biol. 2023 Jul 13;21:158. doi: 10.1186/s12915-023-01655-6 (PMC10347870; doi:10.1186/s12915-023-01655-6)
Supplement: Supplementary file 1 — Additional file 1: Fig. S1. Mutations T323A, M324A and R325A impaired the MUN-catalyzed transition from Munc18-1/Syx1 to the SNARE complex. Representative native gel (upper) shown was from one of three replicates. Quantification of the integrated densities of Munc18-1/Syx1 bands is shown below the native gel. Data are presented as mean values ± SD, n = 3. Fig. S2. Peak elution volume analysis of Munc18-1 WT and mutants in the presence and absence of Syx1. All analyses were performed by size-exclusion chromatography on a Superdex 200 increase 10/300 GL column. Fig. S3. Expression of Munc18-1 TMR in cultured cortical neurons. (A) Quantification of Munc18-1 expression by western blot in neurons infected with control lentivirus (Control) or lentiviruses expressing Munc18-1 shRNA alone (None) or together with wild-type Munc18-1 (WT) or Munc18-1 TMR mutants. Representative results displayed are from one of three independent replicates. (B) Quantitative analysis of the integrated densities of Munc18-1/β-actin bands. The mean values ± SD are shown, n = 3. Fig. S4. The Munc18-1 TMR mutant does not alter synapse formation or the localization of Munc18-1 targeting to synapses in cultured cortical neurons. (A) Cultured cortical neurons infected with control lentivirus or with lentivirus expressing the Munc18-1 shRNA only (None), or together with wild-type Munc18-1 (WT) or the TMR mutant. Neurons were fixed and labeled by double immunofluorescence using Synapsin-1 (to mark synapses) and Munc18-1 antibodies along with fluorescently labeled secondary antibodies (see Materials and Methods). (A) Summary graphs of Synapsin-1-specific fluorescence intensities for all conditions as described above. (B) Summary graphs of ΔF (Fsynapse− Fnon-synapse) of Munc18-1-specific fluorescence intensity for all conditions as described above. Summary graphs of synapse number (C) and size (D), which were quantified from Synapsin-1-specific puncta. Data are presented as mean values ± SEM. Neurons anal [file 12915_2023_1655_MOESM1_ESM.docx]

**Additional file 1: Fig. S1**


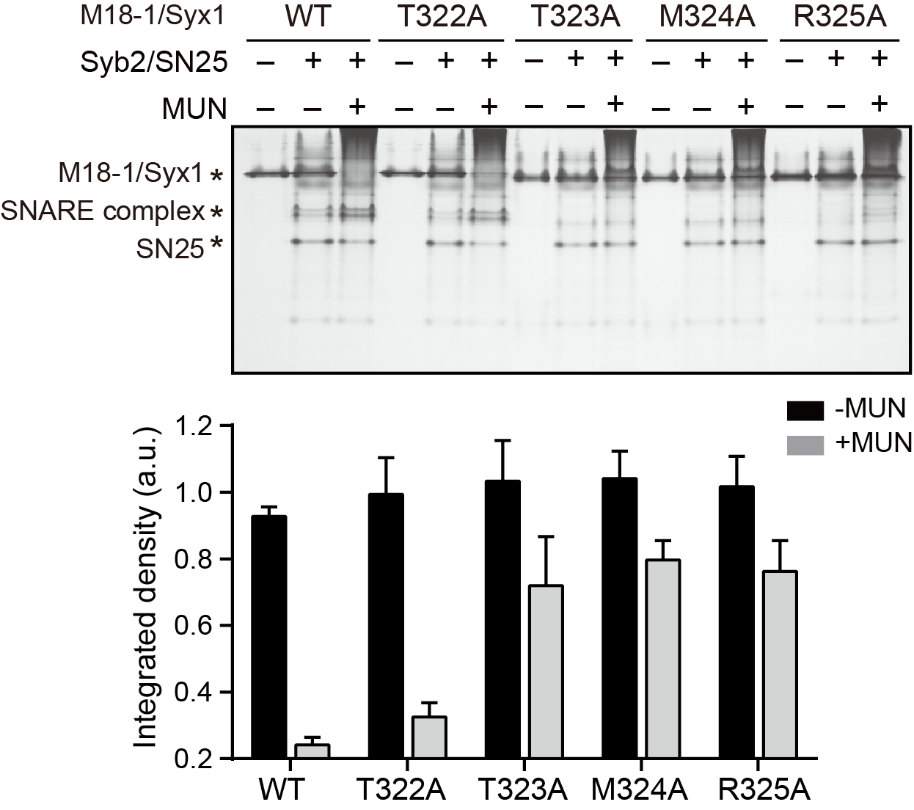


**Fig. S1.** **Mutations T323A, M324A and R325A impaired the MUN-catalyzed transition from Munc18-1/Syx1 to the SNARE complex**. Representative native gel (upper) shown was from one of three replicates. Quantification of the integrated density of Munc18-1/Syx1 bands was shown below the native gel. Data are presented as mean values ± SD, n = 3.

**Additional file 1: Fig. S2**


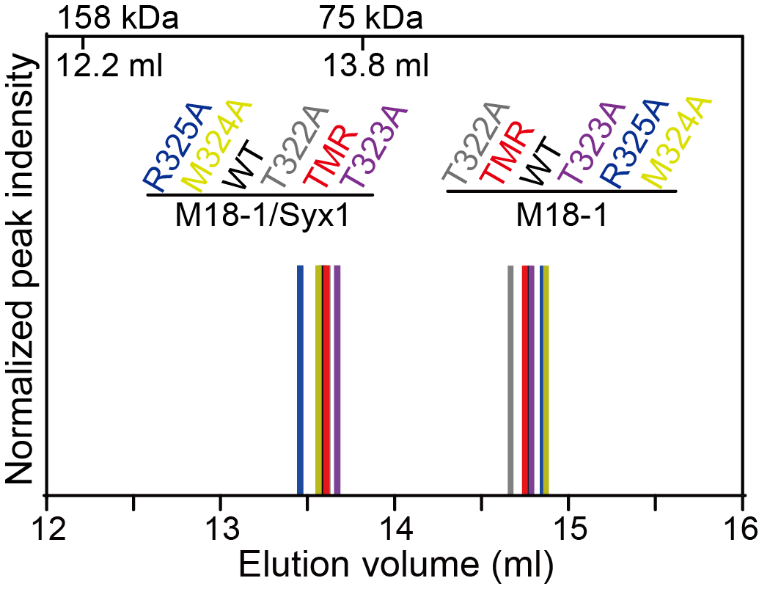


**Fig. S2.** **Peak elution volume analysis of Munc18-1 WT and mutants in the presence and absence of Syx1**. All analyses were performed by size-exclusion chromatography on a Superdex 200 increase 10/300 GL column.

**Additional file 1: Fig. S3**


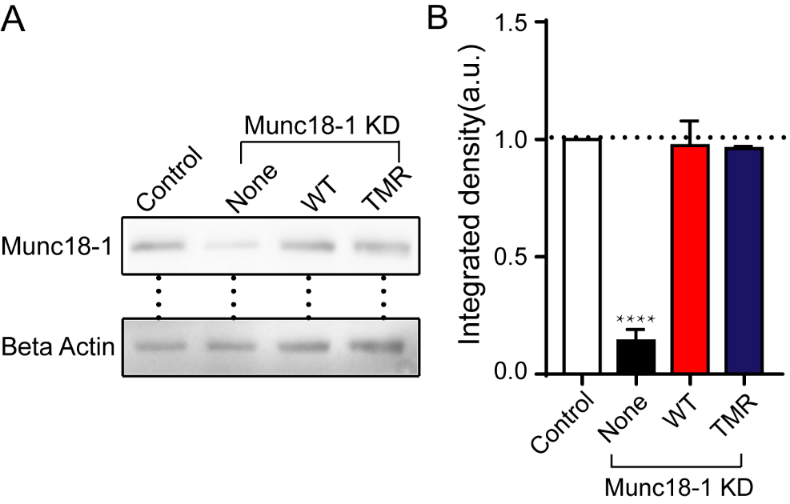


**Fig. S3. Expression of Munc18-1 TMR in cultured cortical neurons.** (A) Quantification of the Munc18-1 expression by western blot in neurons infected with control lentivirus (Control) or lentiviruses expressing the Munc18-1 shRNA alone (None) or together with wild-type Munc18-1 (WT) or Munc18-1 TMR mutants. Representative results displayed are from one of three independent replicates. (B) Quantitative analysis of the integrated densities of Munc18-1/β-actin bands. Shown are mean values ± SD, n = 3.

**Additional file 1: Fig. S4**


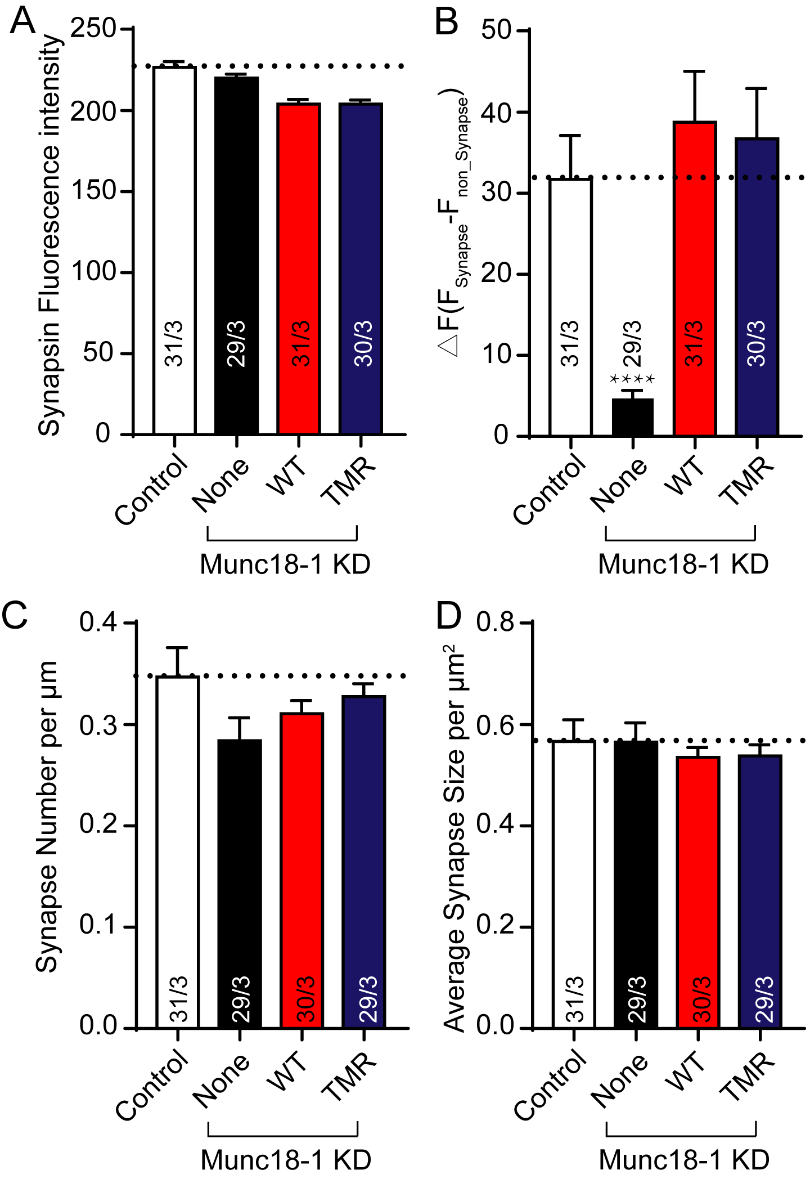


**Fig. S4. The Munc18-1 TMR mutant do not alter synapse formation and the localization of Munc18-1 targeting to synapse in cultured cortical neurons.** (A) Cultured cortical neurons infected with control lentivirus or with lentivirus expressing the Munc18-1 shRNA only (None), or together with wild-type Munc18-1 (WT), or TMR mutant. Neurons were fixed and labeled by double immunofluorescence using Synapsin-1 (to mark synapses) and Munc18-1 antibodies along with fluorescently labeled secondary antibodies (see Materials and Methods). (A) Summary graphs of Synapsin-1-specific fluorescence intensities for all conditions as described above. (B) Summary graphs of ΔF (F_synapse_ − F_non-synapse_) of Munc18-1-specific fluorescence intensity for all conditions as described above. Summary graphs of synapse number (C) and size (D) which were quantified from Synapsin-1-specific puncta. Data are presented as mean values ± SEM. Neurons analyzed are from three independent cultures. Numbers of cells/independent cultures analyzed are listed in the bars. Statistical assessments were performed by the student’s *t* test comparing each condition to the indicated control experiment (****, *P* < 0.0001).

**Additional file 1: Fig. S5**


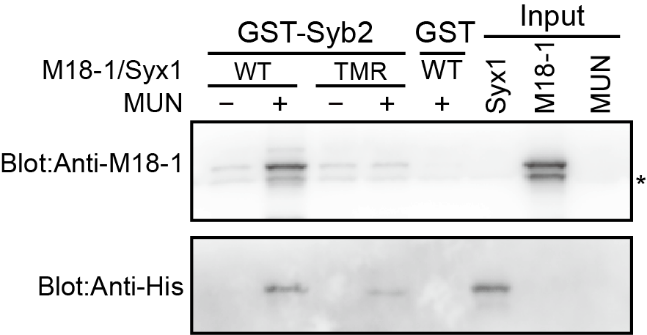


**Fig. S5. Interaction between Munc18-1/Syx1 and Syb2 in the presence of MUN domain analyzed by western blot.** Immunoblotting data was shown with Munc18-1 monoclonal antibody and His-Tag Monoclonal antibody to exhibit the binding of Munc18-1 and Syx1 respectively. Bands of Munc18-1 [degradation](javascript:;) was indicated with Asterisk. The degradation is common in Munc18-1 and has no effect on Syx1 binding. Representative results displayed are from one of three independent replicates.
