# Supplementary figures and images for: Identification of residues critical for the extension of Munc18-1 domain 3a
Source: BMC Biol. 2023 Jul 13;21:158. doi: 10.1186/s12915-023-01655-6 (PMC10347870; doi:10.1186/s12915-023-01655-6)

Figure 1B

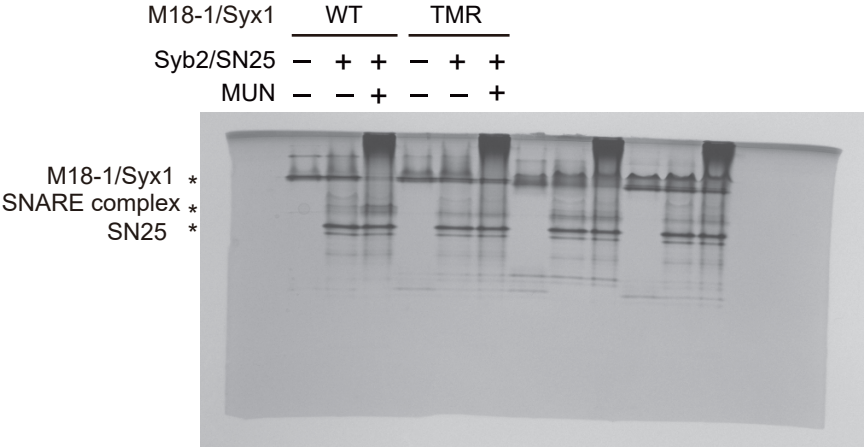

Figure 1D

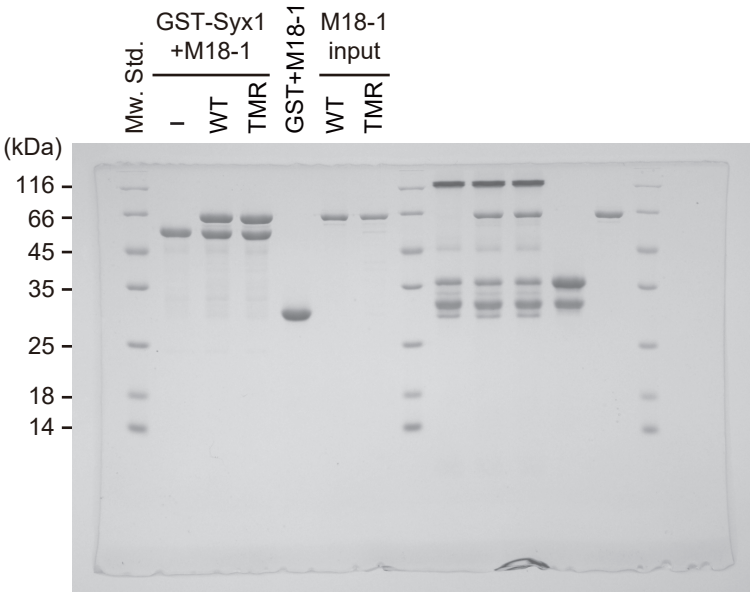

Figure 4D

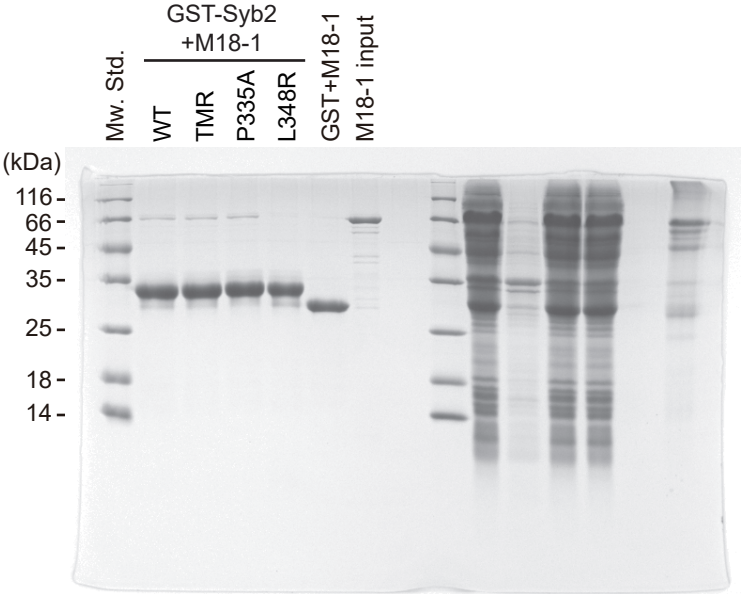

Figure 4F

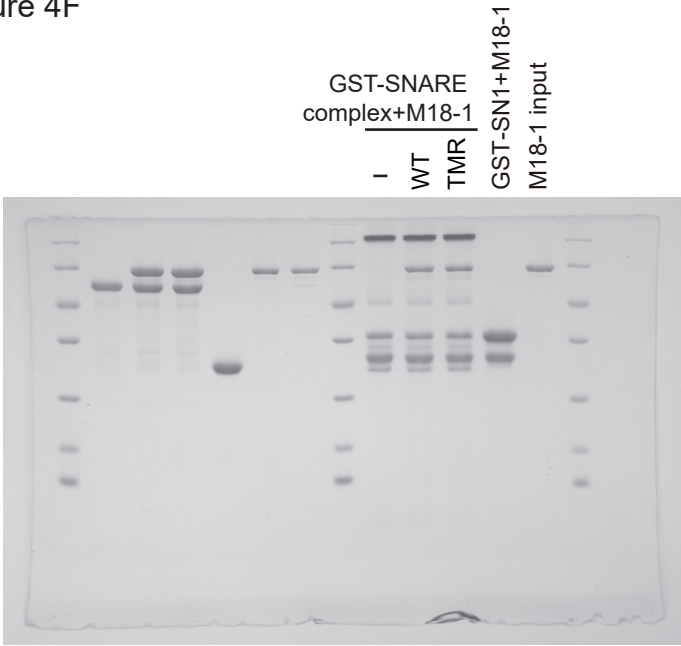

Figure 5C

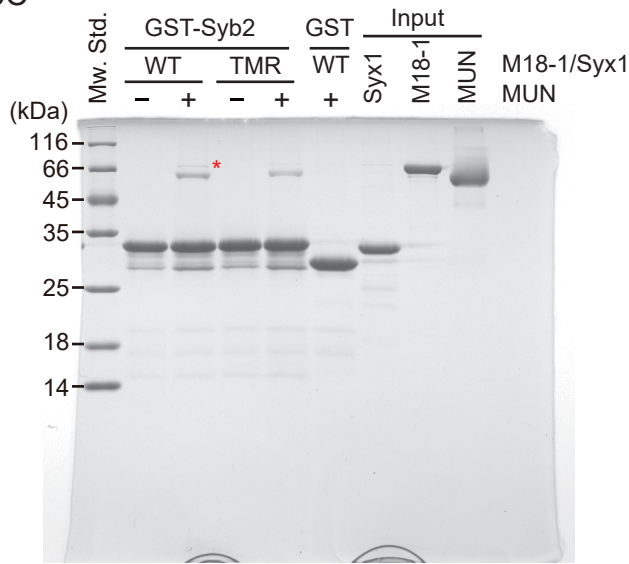

Figure S1

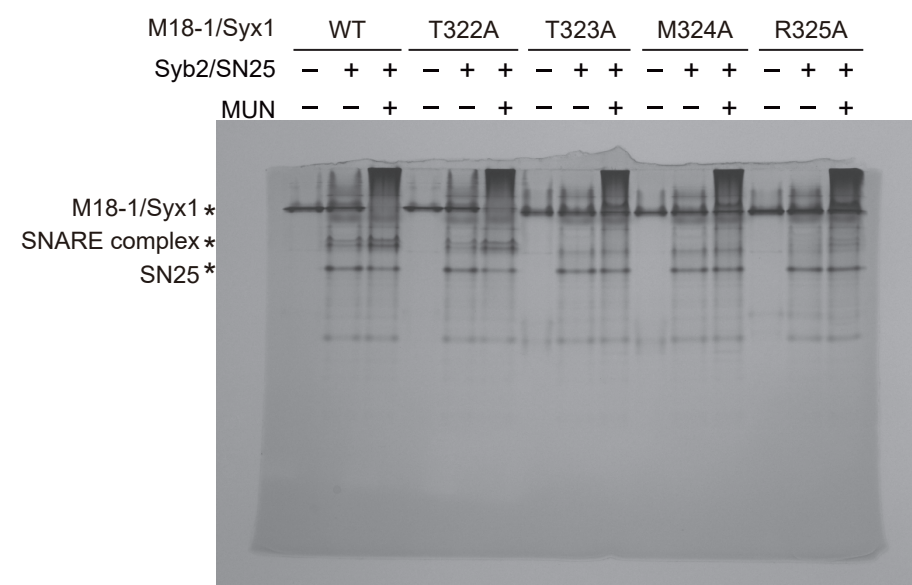

Figure S3

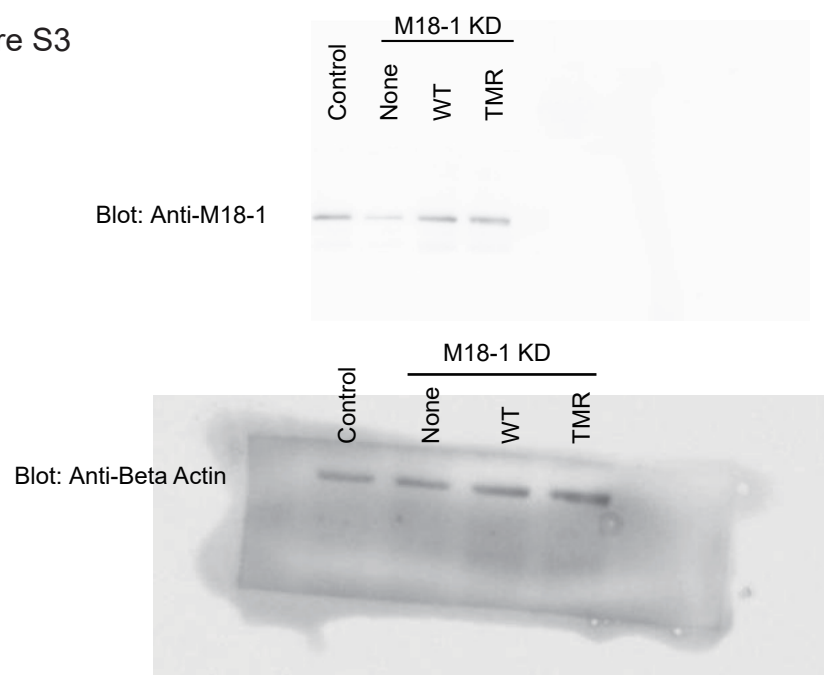

Figure S5

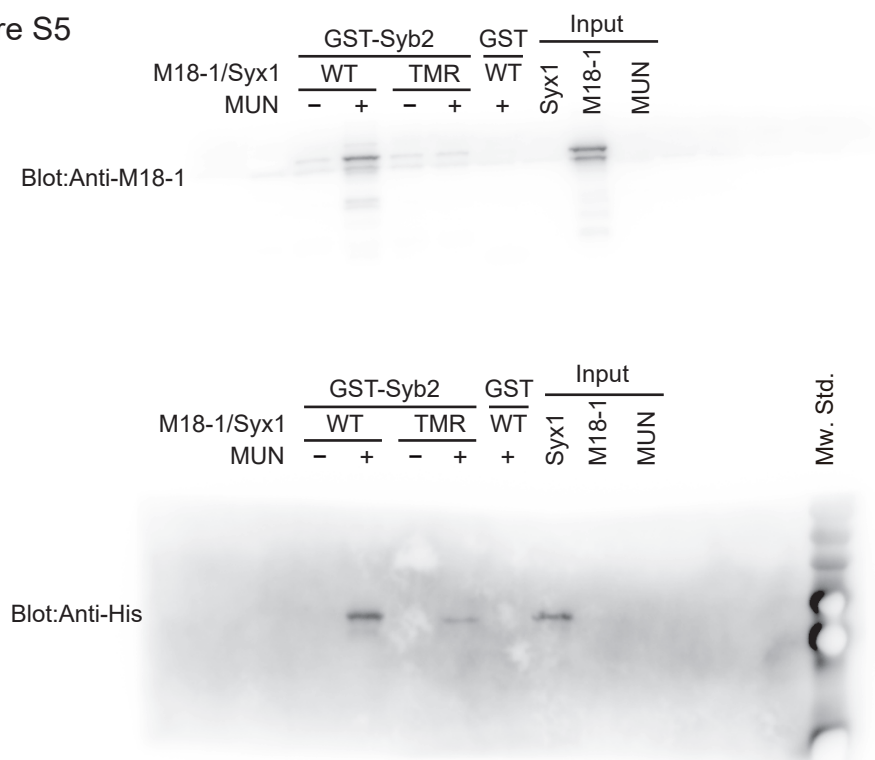

Supplement: Supplementary file 2 — Additional file 2. Uncropped images of gels and Western blots shown in this paper. [file 12915_2023_1655_MOESM2_ESM.pdf]
