# Supplementary material for: Identification of residues critical for the extension of Munc18-1 domain 3a
Source: BMC Biol. 2023 Jul 13;21:158. doi: 10.1186/s12915-023-01655-6 (PMC10347870; doi:10.1186/s12915-023-01655-6)
Supplement: Supplementary file 4 — Additional file 4. Original images of confocal imaging for Fig. 3A and B. [file 12915_2023_1655_MOESM4_ESM.pdf]

mCherry-Syx1

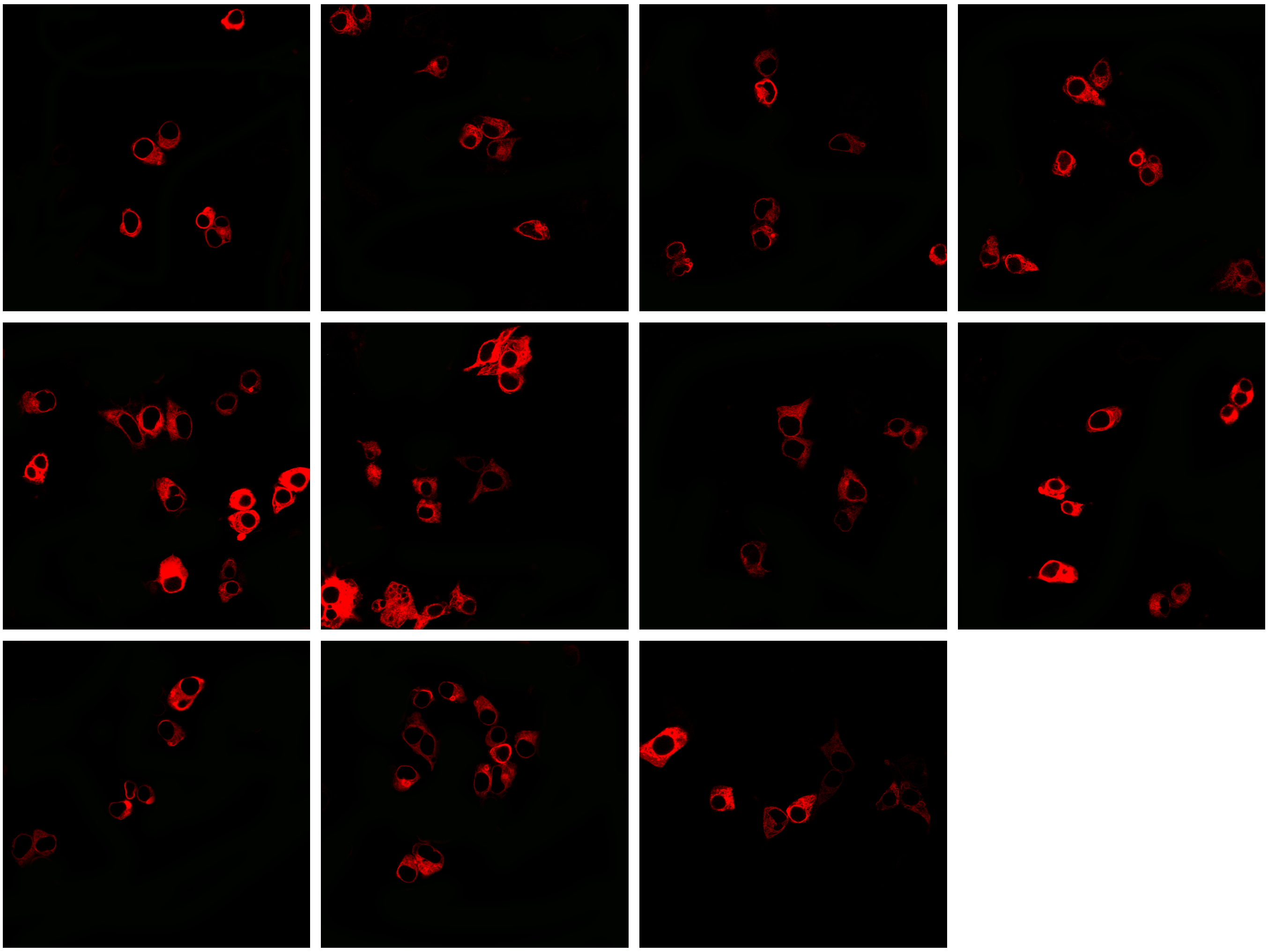

Munc18-1-EGFP

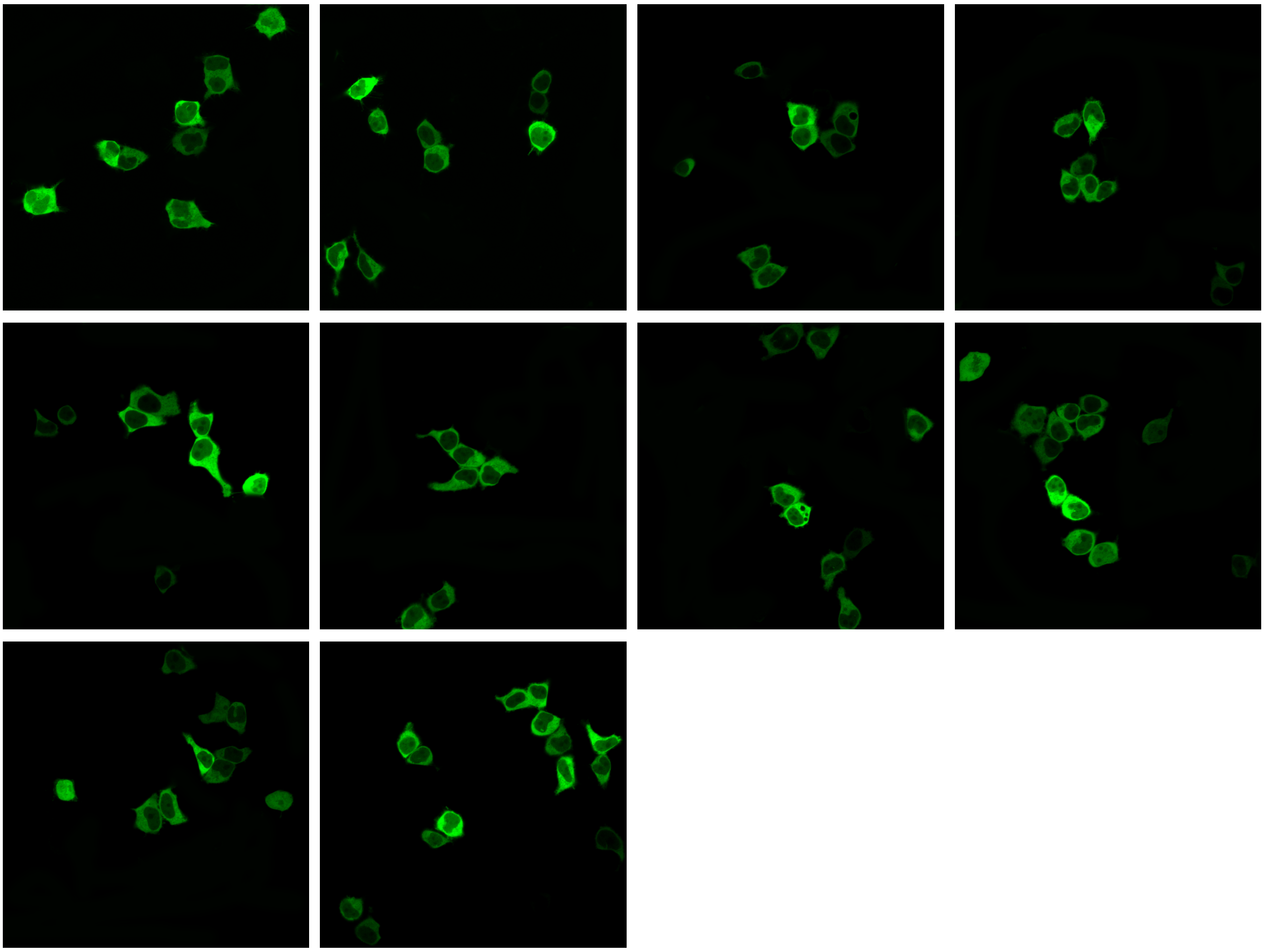

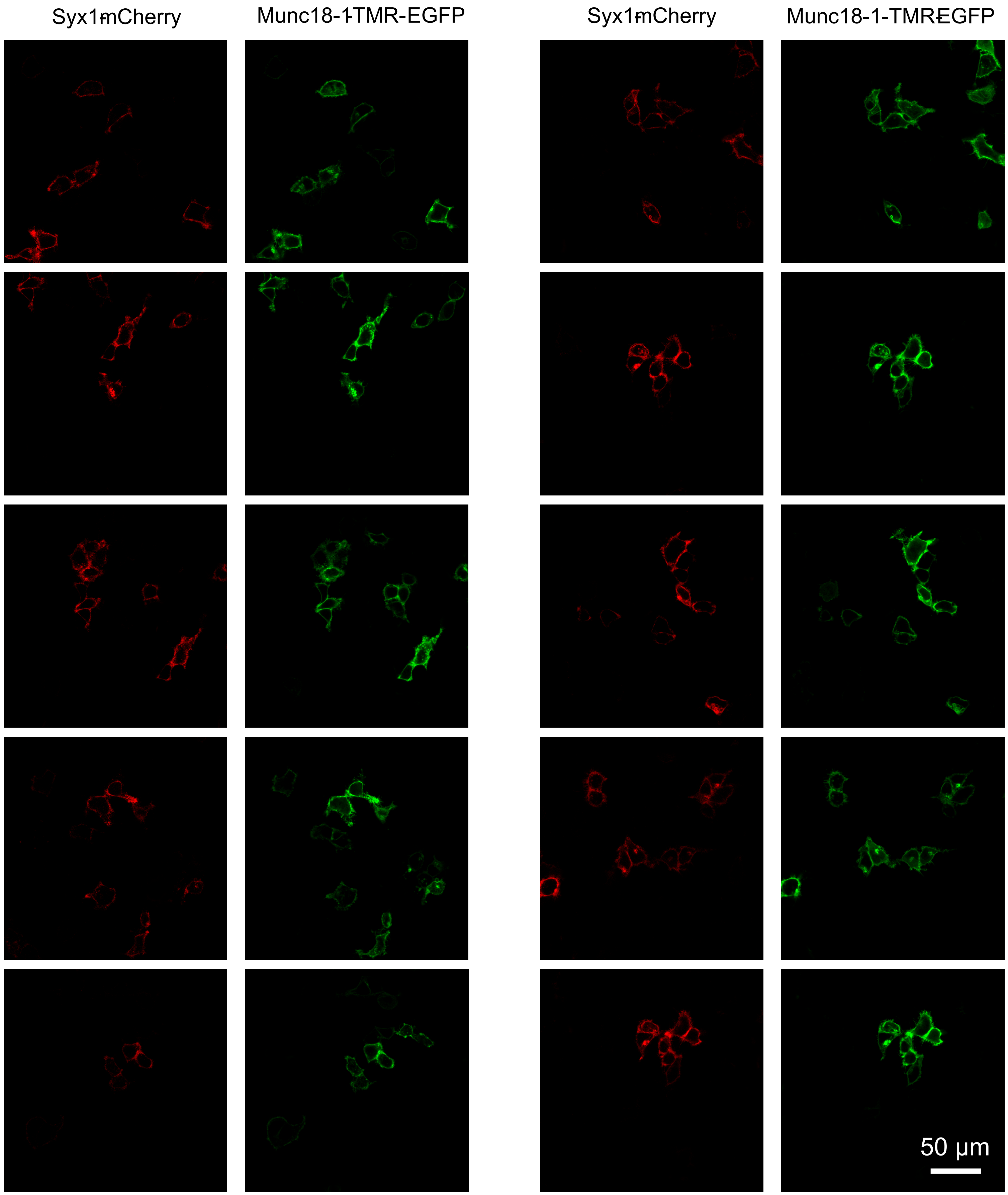
Syx1-mCherry
Munc18-1-TMR-EGFP
Syx1-mCherry
Munc18-1-TMR-EGFP
50 μm

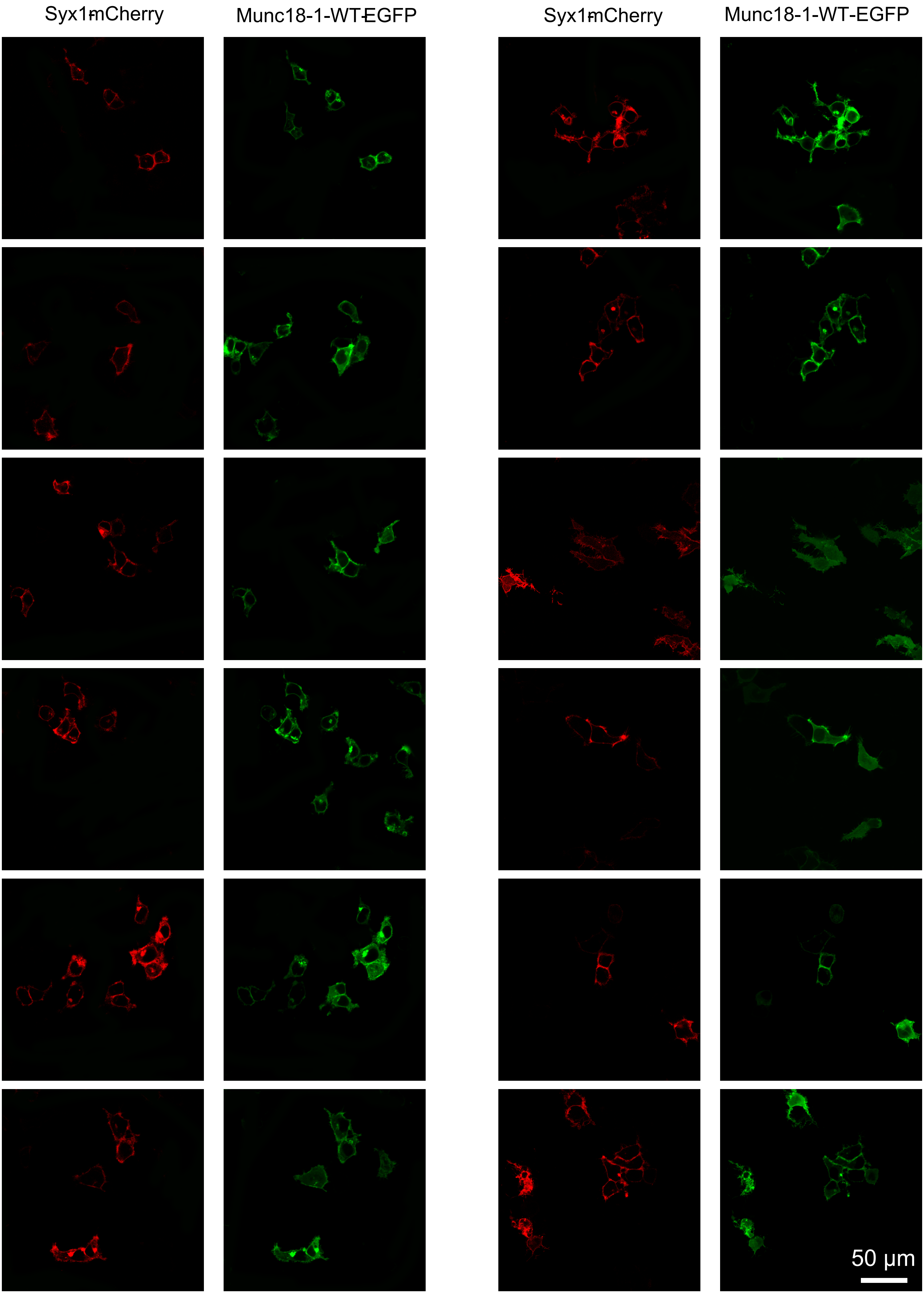

The image resolution is 1024*1024 pixel, 0.21 μm per pixel. Scale bar is applied to all the images
